# Using the community-based breeding program (CBBP) model as a collaborative platform to develop the African Goat Improvement Network—Image collection protocol (AGIN-ICP) with mobile technology for data collection and management of livestock phenotypes

**DOI:** 10.3389/fgene.2023.1200770

**Published:** 2023-09-06

**Authors:** M. Jennifer Woodward-Greene, Jason M. Kinser, Heather J. Huson, Tad S. Sonstegard, Johann Soelkner, Iosif I. Vaisman, Paul Boettcher, Clet W. Masiga, Christopher Mukasa, Solomon Abegaz, Morris Agaba, Sahar S. Ahmed, Oliver F. Maminiaina, Tesfaye Getachew, Timothy N. Gondwe, Aynalem Haile, Yassir Hassan, Absolomon Kihara, Aly Kouriba, Hassan A. Mruttu, Denis Mujibi, Wilson Nandolo, Barbara A. Rischkowsky, Benjamin D. Rosen, Brian Sayre, Maria Taela, Curtis P. Van Tassell

**Affiliations:** ^1^ National Agricultural Library, USDA Agricultural Research Service, Beltsville, MD, United States; ^2^ Animal Genomics Improvement Laboratory, USDA Agricultural Research Service, Beltsville, MD, United States; ^3^ Bioinformatics and Computational Biology Program, School of Systems Biology, College of Science, George Mason University, Manassas, VA, United States; ^4^ School of Physics, Astronomy, and Computational Sciences, College of Science, George Mason University, Fairfax, VA, United States; ^5^ Department of Animal Science, College of Agriculture and Life Sciences, Cornell University, Ithaca, NY, United States; ^6^ Acceligen Inc., Eagan, MN, United States; ^7^ Department of Sustainable Agricultural Systems, Division of Livestock Sciences, BOKU—University of Natural Resources and Life Sciences, Vienna, Austria; ^8^ Food and Agriculture Organization of the United Nations, Animal Production and Health Division, Rome, Italy; ^9^ Association for Strengthening Agricultural Research in Eastern and Central Africa (ASARECA), Entebbe, Uganda; ^10^ National Animal Genetic Resource Centre and Data Bank, Entebbe, Uganda; ^11^ Ethiopian Institute of Agricultural Research, Addis Ababa, Ethiopia; ^12^ Nelson Mandela African Institution of Science and Technology, Arusha, Tanzania; ^13^ Cell Biology Department, Biotechnology Research Institute, National Research Centre, Giza, Egypt; ^14^ Department of Zootechnical, Veterinary and Piscicultural Research (DRZVP), National Center for Applied Research in Rural Development (CENRADERU), Antananarivo, Madagascar; ^15^ International Center for Agricultural Research in the Dry Areas (ICARDA), Addis Ababa, Ethiopia; ^16^ Department of Animal Science, Lilongwe University of Agriculture and Natural Resources, Lilongwe, Malawi; ^17^ Department of Animal Genetic Resources Development, Animal Production Research Center, Ministry of Animal Resources, Khartoum North, Sudan; ^18^ International Livestock Research Institute, Nairobi, Kenya; ^19^ Institut d’Économie Rurale, Bamako, Mali; ^20^ Ministry of Livestock and Fisheries, Dodoma, Tanzania; ^21^ Department of Biology, Virginia State University, Petersburg, VA, United States; ^22^ Agrarian Research Institute of Mozambique, Directorate of Animal Science, Maputo, Mozambique

**Keywords:** image analysis, phenotype, body weight, coat, color, one health, FAMACHA, tooth age

## Abstract

**Introduction:** The African Goat Improvement Network Image Collection Protocol (AGIN-ICP) is an accessible, easy to use, low-cost procedure to collect phenotypic data via digital images. The AGIN-ICP collects images to extract several phenotype measures including health status indicators (anemia status, age, and weight), body measurements, shapes, and coat color and pattern, from digital images taken with standard digital cameras or mobile devices. This strategy is to quickly survey, record, assess, analyze, and store these data for use in a wide variety of production and sampling conditions.

**Methods:** The work was accomplished as part of the multinational African Goat Improvement Network (AGIN) collaborative and is presented here as a case study in the AGIN collaboration model and working directly with community-based breeding programs (CBBP). It was iteratively developed and tested over 3 years, in 12 countries with over 12,000 images taken.

**Results and discussion:** The AGIN-ICP development is described, and field implementation and the quality of the resulting images for use in image analysis and phenotypic data extraction are iteratively assessed. Digital body measures were validated using the PreciseEdge Image Segmentation Algorithm (PE-ISA) and software showing strong manual to digital body measure Pearson correlation coefficients of height, length, and girth measures (0.931, 0.943, 0.893) respectively. It is critical to note that while none of the very detailed tasks in the AGIN-ICP described here is difficult, every single one of them is even easier to accidentally omit, and the impact of such a mistake could render a sample image, a sampling day’s images, or even an entire sampling trip’s images difficult or unusable for extracting digital phenotypes. Coupled with tissue sampling and genomic testing, it may be useful in the effort to identify and conserve important animal genetic resources and in CBBP genetic improvement programs by providing reliably measured phenotypes with modest cost. Potential users include farmers, animal husbandry officials, veterinarians, regional government or other public health officials, researchers, and others. Based on these results, a final AGIN-ICP is presented, optimizing the costs, ease, and speed of field implementation of the collection method without compromising the quality of the image data collection.

## Introduction

The African Goat Improvement Network Image Collection Protocol (AGIN-ICP) was developed systematically over a 3-year period in conjunction with the AdaptMap project (Stella et al., 2018) and the African Goat Improvement Network (AGIN) (USDA, 2020). These are coordinated, multi-national efforts to characterize, evaluate, and conserve goat population genetic resources globally, and in Africa respectively. This paper describes the development of AGIN-ICP as a case study in the application of the AGIN collaboration model working directly with community-based breeding programs (CBBP) for multi-level (farmers and local students, animal husbandry officials, junior and seasoned researchers) and multi-national capacity development in human, and technological resources in the developing and the developed worlds (Van Tassell et al., 2023). Images collected in the last stage of AGIN-ICP development were used to establish the Precise Edge Image Segmentation Algorithm (PE-ISA) and software which was used to validate that digital phenotypes could be extracted from AGIN-ICP collected images that reflected accurate phenotypic body measures for height (0.931), length (0.943), and girth 0.893) measures (Woodward-Greene et al., 2022).

A major objective of the AGIN collaborative model is that it is led from the community level. Farmers and students, when guided by animal husbandry officials, researchers, and other specialists, are critical to finding original, yet practical solutions. Each AGIN participant, therefore, has a stake and an important role to play in innovation. Individually, that has a rallying effect—that through mutual respect and a sincere need for all perspectives—gives energy and purpose to the work. The development of digital livestock phenotyping provided an opportunity for many AGIN participants to develop, experience, and discover cutting edge technology. Researchers in 12 sampling teams employed the collection method in 11 African countries, in addition to the US, sampling approximately 2,000 goats and collecting over 12,000 images.

**FIGURE 1:**
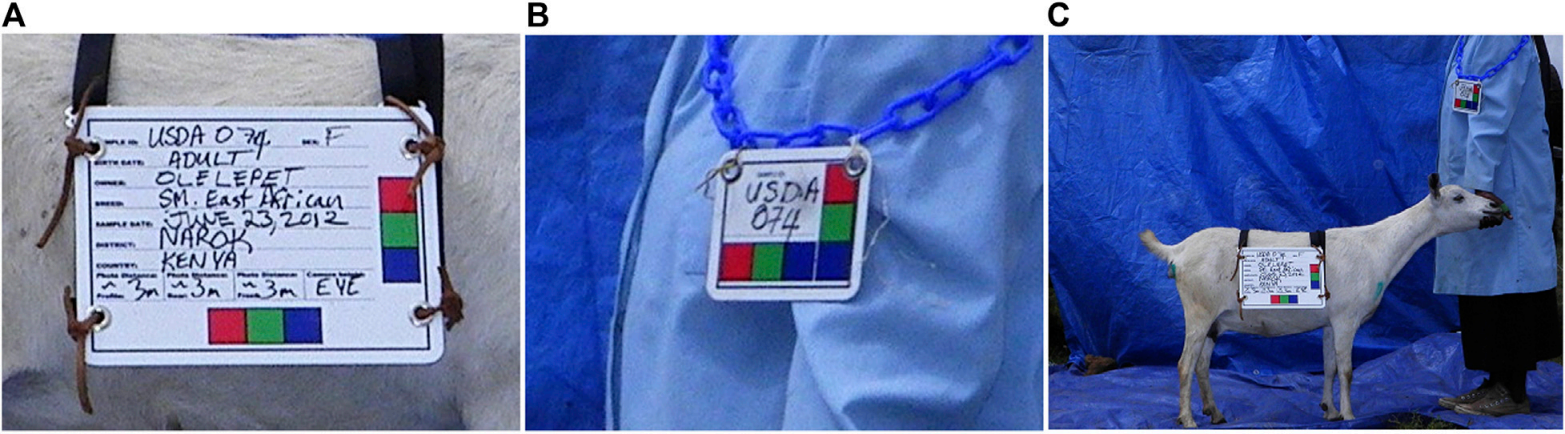
Novel calibration sign and harness **(A,C)**, and sample identifier sign **(B)** for AGIN-ICP images.

The purpose of AGIN-ICP and the testing was to determine if sufficiently high-quality digital images could be collected under field sampling conditions such that accurate phenotype data could be extracted from the images. The first draft, original protocol method, included five poses: 1) rear view, 2) sign view (Figure 1C), 3) front view, 4) teeth close-up, and 5) FAMACHA (eye) close-up. Specialized equipment included three novel calibration signs to include in the image - one to hang from the goat’s back and two smaller ones to hang from the neck of the goat and the animal handler, respectively. In the sign view, the right side of the animal is photographed (animal head facing to the right in the image), so the (often protruding) rumen on the left-side does not interfere with digital measurements. The iterative process with AGIN collaborators focused on the following three areas to optimize collection procedures and image quality for data extraction:

1. The method itself (posing, set-up),
2. Communication with—and training of—sampling teams, and
3. Development and refinement of the photo sampling equipment and sampling kit.

As the protocol and its evolution are described in detail in this paper, one may be struck with the simplicity of the tasks involved and question the level of detail included here. However, the iterative development of the final AGIN-ICP and the constant review of images coming from the field that drove most of the changes, show that these details are easy to overlook. It is also easy to misinterpret how these seemingly obvious steps need to be performed in a precise manner, or why they are critical for ultimate image quality. Sampling teams, and in particular the photographer, must remain focused on the many seemingly minor details as the sampling days wear on and vigilant that all procedures are completed with precision and attention to detail. A lack of attention to these details, as we have seen in the development of AGIN-ICP process, can make rendering the images captured much more difficult and potentially unusable for digital phenotype extraction. Considering the expense in time, equipment, travel, and logistical planning required for sampling trips, it is imperative that sampling teams understand the rationale for each step, the correct manner of performing it, and the ultimate purpose to ensure the highest quality data collection. To that end, we include examples of the images after extraction—which were not available to sampling teams throughout the stages of AGIN-ICP development.

### Global food security

An important goal for CBBPs is to breed resilient, productive food animals. This will enhance food security and income of African goat farmers. Two key aspects of this goal are 1) establishing sustainable and efficient production and health management systems, and 2) identifying, conserving, and selecting traits that ensure productivity, disease and parasite resistance, and adaptability to climate change and other stressors. This will allow farmers to provide high-quality nutritious food for their families and generate income. Collection of phenotypic data is considered critically important by the Food and Agriculture Organization (FAO) of the United Nations to further animal genetic resources characterization and conservation (Commission on Genetic Resources for Food and Agriculture, 2007). The FAO specifically notes the critical need for consistent collection methods of phenotypic data across animal populations (FAO, 2011). This consistency in capturing phenotypes is required to inform genomic science in the research and development of state-of-the-art genomics tools for genome to phenome prediction. This digital phenotype collection method may enable even the poorest countries to take advantage of this advancing science. It may ultimately identify and conserve their most important adapted animal genetic resources. The AGIN-ICP includes images to collect phenotypic data on health status predictions for anemia, weight, age, and coat color and pattern. It may also have applications in the One Health (One Health, 2023) approach to public health, which considers the connection between animal and human public health in disease outbreaks. One Health identifies zoonotic disease surveillance in animal populations as an important tool in preventing human disease outbreaks (World Bank, 2012; One Health, 2023). Phenotype and health data may be collected using the AGIN-ICP. It can be accumulated in regional or global data repositories for open data sharing by researchers and health officials. The original user (farmer or veterinarian) may also have access for animal record keeping or real-time decisions on disease status or treatment, production, nutrition, and breeding, etc.

### Body weight prediction

Weight prediction from images is not a new concept (Phillips and Dawson, 1936; Schofield et al., 1999; Ozkaya, 2013). Body weight is important for many decisions in livestock health, production, and marketing (Mahieu et al., 2011). The expense and inconvenience of using livestock scales to record weights have fueled decades of research into alternative methods to obtain reasonably accurate values (Muhammad et al., 2006; Mahieu et al., 2011; Takaendengan et al., 2012). Weight gain is dependent on age, stage of lactation or gestation, nutritional or disease status, and breed (World Bank, 2012), and may inform breeding and production decisions. In genomics and genomic tool development, physical measurements such as size, shape, and coat color and pattern, can be associated with productivity or with adaptive genes for traits such as milk production, fertility, disease and parasite resistance, or growth rate. Animals are often priced in the marketplace by weight or age. Using a scale to measure body weight is the most consistent and accurate method. However, many producers, in particular goat or sheep producers or those in developing countries, do not have ready access to scales (Abegaz and Awgichew, 2009; Mahieu et al., 2011). The least expensive scales are hanging or bathroom scales. These devices are cumbersome to use because they require lifting or holding the animal, making those scales useful for smaller animals only. Alternatively, physical measurements of size have long been used to estimate weight cheaply. Several formulas have been developed and tested for accuracy on particular animal types or breeds (Muhammad et al., 2006; Sowande and Sobola, 2008; Abegaz and Awgichew, 2009; Ozkaya et al., 2009; Mahieu et al., 2011; Takaendengan et al., 2012). Weight prediction formulas generally use some combination of chest girth (CG), body length (BL), and/or height at the withers (HW) to predict body weight (BW) (Abegaz et al., 2013; Horner, 2021). Body measurements are taken with calipers (Touchberry and Lush, 1950; Calipers, 2014) or taken with a cloth measuring tape that is either designed for sewing or designed specifically as a goat weigh tape with predicted weights (based on chest girth) printed on the tape. Conversion tables are available online for producers to predict body weights based on chest girth measures (Campbell, 2002; Bar None Meat Goats, 2021; Horner, 2021).

### Teeth to determine animal age and health

Tooth age is a long established method used to estimate the live-animal-age or age-at-death of an animal based on permanent tooth eruption (ARC, 1999; Greenfield and Arnold, 2008; Matika et al., 1992; Fias Co Farm, 2023). In livestock operations, the best method to determine an animal’s age is by keeping accurate individual birth records. However, in many operations, especially in limited resource areas, records may be incomplete or altogether unavailable (Timon, 1992; Ephrem, 2013). For animal groups without birth records, age can be estimated by examining the teeth to identify the number of adult teeth erupted (Oltenacu and Stanton, 1999; Soltero-Rivera, 2022; Fias Co Farm, 2023). While not exact, tooth age estimation is a relatively quick and easy method for farmers and veterinarians to approximate the productive stage of an animal, i.e., growth (mostly deciduous or milk teeth present), maintenance and breeding age (mostly or all permanent adult teeth present), or expected remaining productive life (amount of wear on adult teeth) (Oltenacu and Stanton, 1999). At a livestock market, tooth age can be used to assess the carcass market value (younger animals are assumed to have higher quality meat) (Matika et al., 1992), or to comply with export requirements (Canadian Food Inspection Agency, 2014). Archeologists use tooth age to determine the age of death of livestock and to infer the type of production systems the animals were reared in. For example, if mainly young animals were slaughtered, leaving lactating females without offspring, it may be assumed that the economy was based on milk production (Baker and Worley, 2019). This assumption could also be employed to characterize and assess current production systems in resource poor regions where animal birth, growth, health, or sales/market records are lacking. Finally, teeth can be an indicator of current or future health. Goats need their teeth to be able to tear the grass as they graze, and an animal with broken teeth may not thrive. This ‘soundness’ of the mouth has long been an observation to determine the health, and value of grazing livestock (eXtension Goat Community of Practice, 2023).

### FAMACHA anemia score

The FAMACHA card is a simple tool developed in South Africa to estimate the level of anemia in sheep and goats by comparing the conjunctiva color of the animal to a series of five color categories associated with a blood anemia values (Malan et al., 2001; Kaplan et al., 2004). It was named after the South African parasitologist, Francois “Fafa” Malan who created it (Comis, 2010). The FAMACHA card is laminated and includes an image of an animal eye to show the proper way to examine the conjunctiva, along with 5 boxes of varying shades of pink to designate the 5 categories of anemia. The FAMACHA method enables producers to identify animals within groups that are most likely infested with worms, as indicated by anemia. Resistance to worming medications is a critical problem in the livestock industry (Leask et al., 2013). By treating only those animals with heavy worm infestation, producers can save time, money, and critically—help to inhibit the development of resistance to anthelmintics (Shoenian, 2023). The FAMACHA method has been validated in numerous studies across many breeds and regions around the world, proving to be effective in sheep and goats in a wide range of climates and production systems (Malan et al., 2001; Vatta et al., 2001; Van Wyk and Bath, 2002; Kaplan et al., 2004; Ejlertsen et al., 2006; Moors and Gauly, 2009; Idika et al., 2012; Sotomaior et al., 2012; Leask et al., 2013).

### Coat color and pattern

Coat color and pattern are important to livestock breeders for the value that preferred animal characteristic coat colors may bring in the market. Preferences may be based on the association of desired production traits such as growth rate, milk production, twinning, or heat tolerance with a particular breed that is also known for its coat color and patterns. Preferences for color also can be cultural, and could include risks for negative selection (selecting for traits that lower production) (Getachew et al., 2020). Color preferences are also often associated with compliance with purebred standards, and directly impact the market value of animals. Coat color has long been studied as a visible breeding objective for livestock (Martin et al., 2016).

### PreciseEdge Image Segmentation Algorithm and software

The objective of developing AGIN-ICP was to generate images that meet the requirements for successfully producing an image analysis process and software capable of extracting accurate phenotypes from digital images. To provide accurate measurements, digital images require software that can identify the parts of the goat to be measured, or features, in the images. This demands the highest possible precision to isolate these features. This challenge led to the development of the PreciseEdge Image Segmentation Algorithm (PE-ISA), developed using images collected in a highly controlled manner in the final stage of AGIN-ICP development, when the protocol iteration was the most mature. This approach allowed for validation that the images could in fact, be used for the digital extraction of phenotypes directly from the images. The AGIN-ICP has been validated by extracting digital body measures from collected images. The correlation between manual body measures and digital measures were found to be high with Pearson correlation coefficients 0.931 for height, 0.943 for length, and 0.893 for girth (Woodward-Greene et al., 2022). Key aspects of the PE-ISA development included processing of input images using portable network graphics (PNG) compression for increased precision. The PreciseEdge Algorithm also reduces user input for image processing, reducing labor costs on the analysis phase of the phenotype collection with AGIN-ICP (Woodward-Greene et al., 2022). Additionally, software needed to deploy the algorithm and provide output data files with digital phenotype measures, as well as labeled images for further analyses, has been developed (*manuscript in process*). This software requires no special facilities or advanced skills; and users need only a laptop and mouse to process collected images. Examples of input and output images from the software, using the PreciseEdge Algorithm, are shown for body measurements in Table 1, and for health phenotypes (teeth and FAMACHA score) in Table 2.

**TABLE 1:** AGIN-ICP Input Images (left). Body size phenotypes extracted using the PreciseEdge Image Segmentation Algorithm embedded in software to record measurements, and mark them on the images (right).


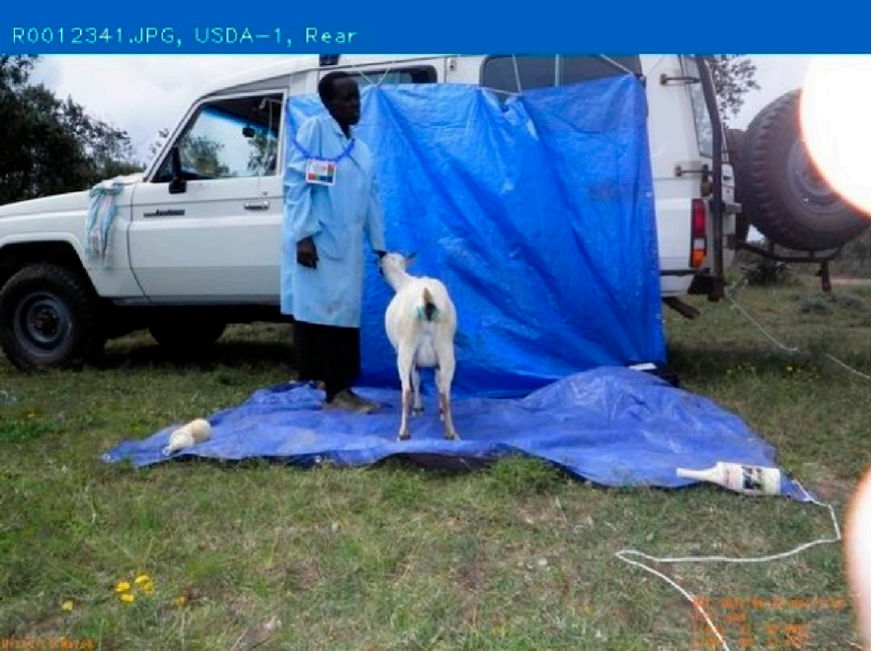	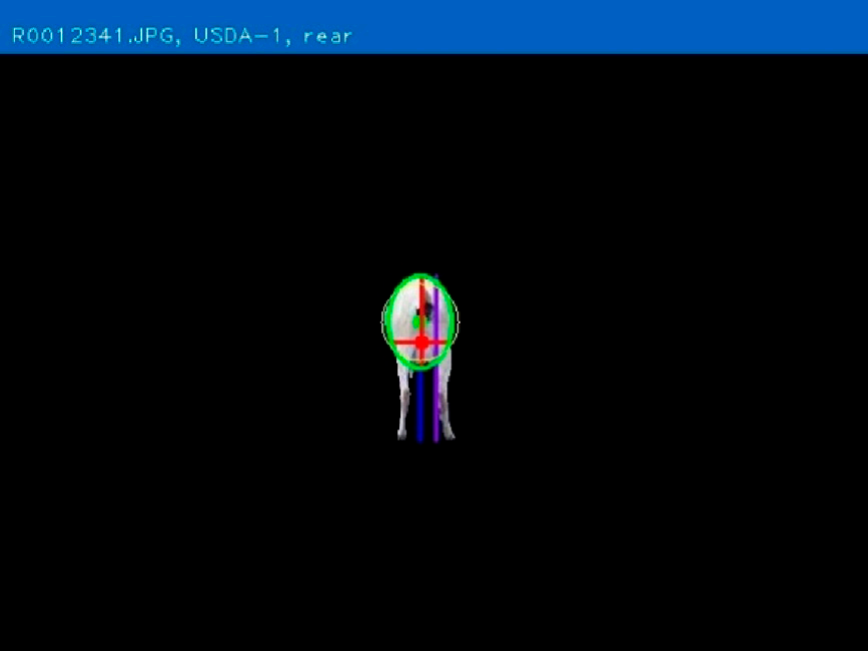
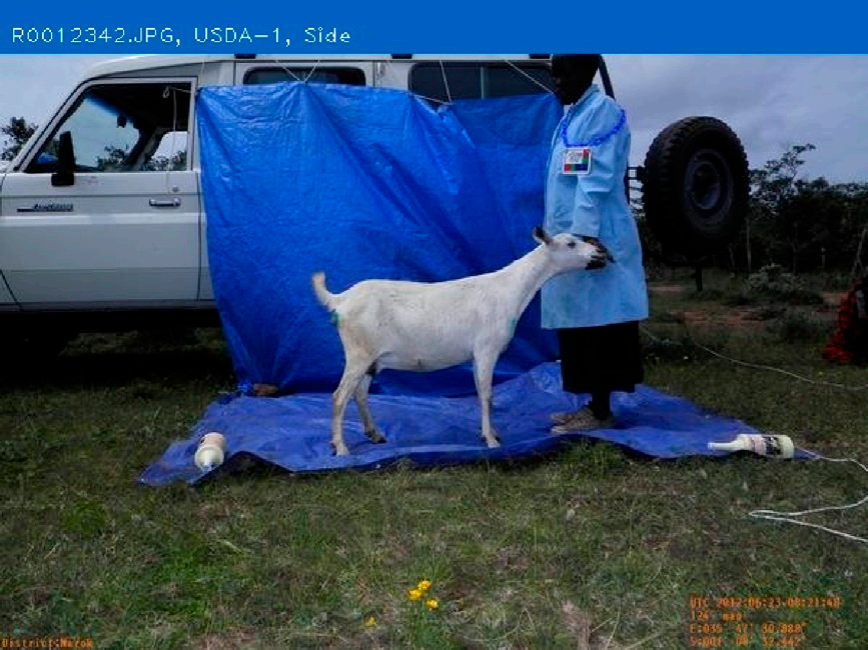	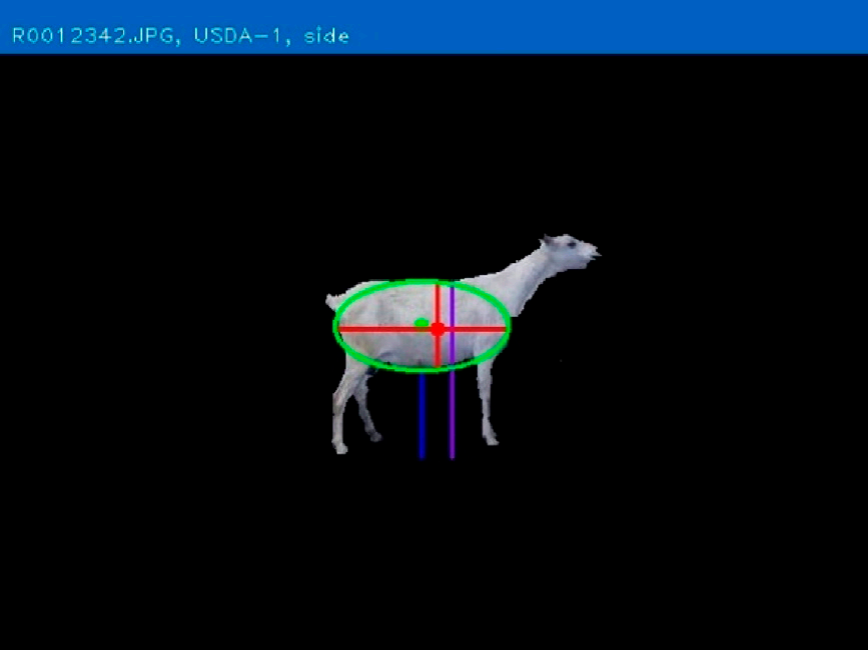
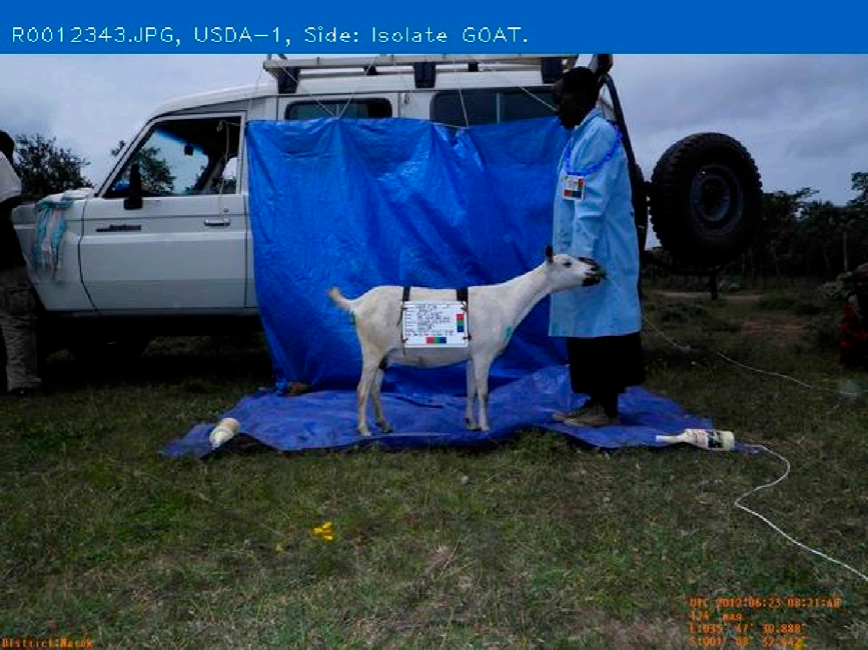	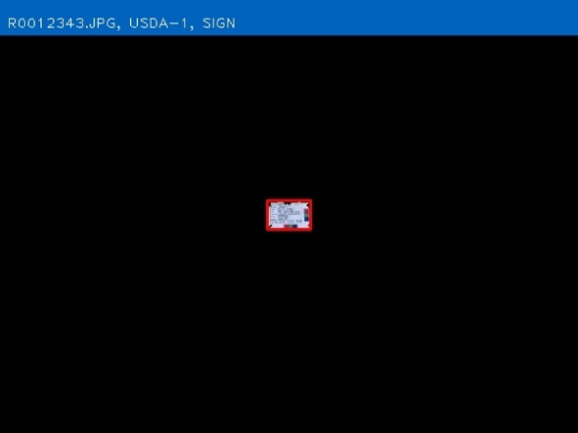

**TABLE 2:** AGIN-ICP Input Images (left). Health (teeth, FAMACHA score) phenotypes extracted using the PreciseEdge Image Segmentation Algorithm embedded in software to record measurements, and mark them on the images (right).


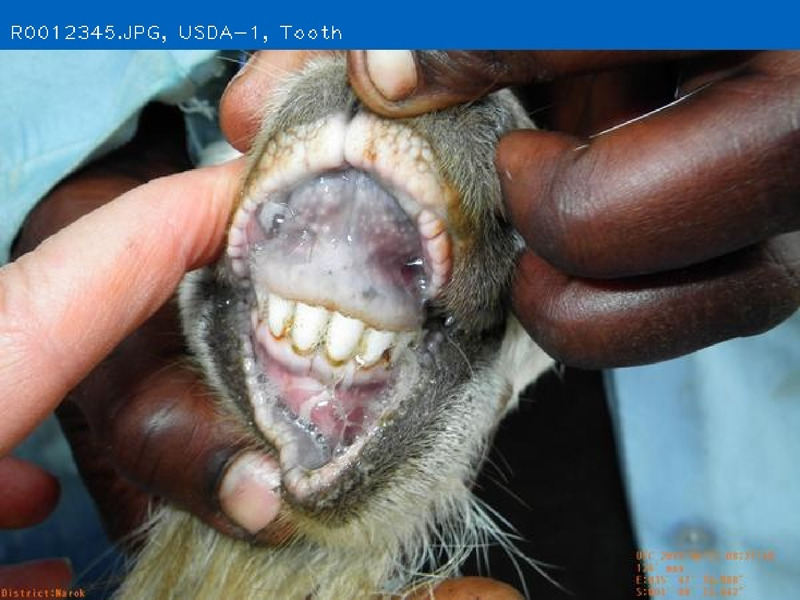	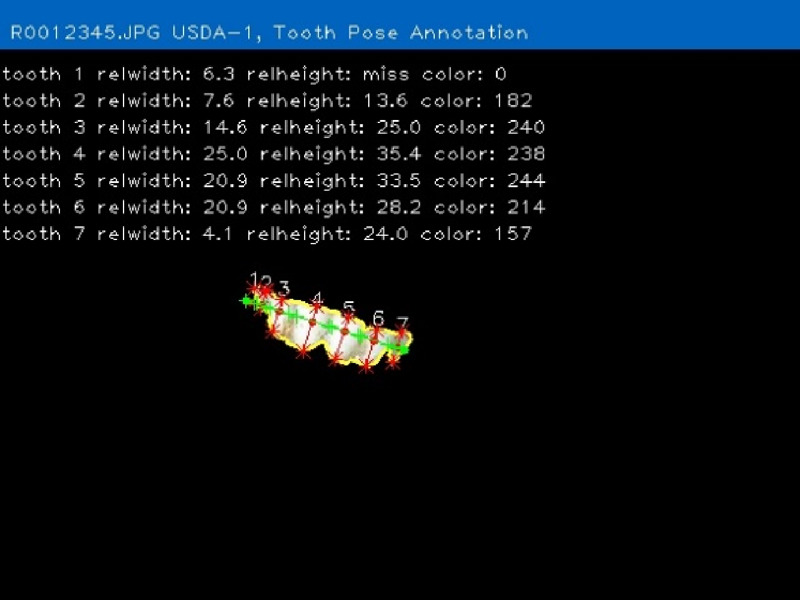
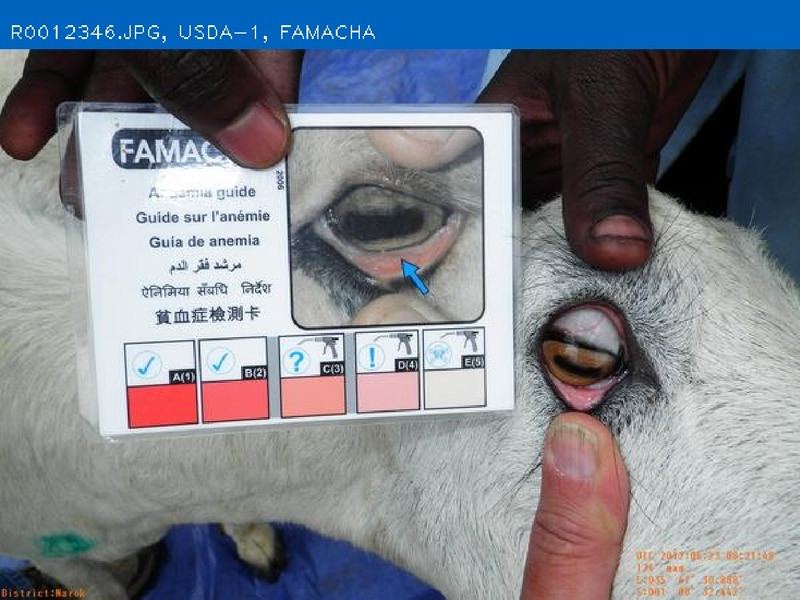	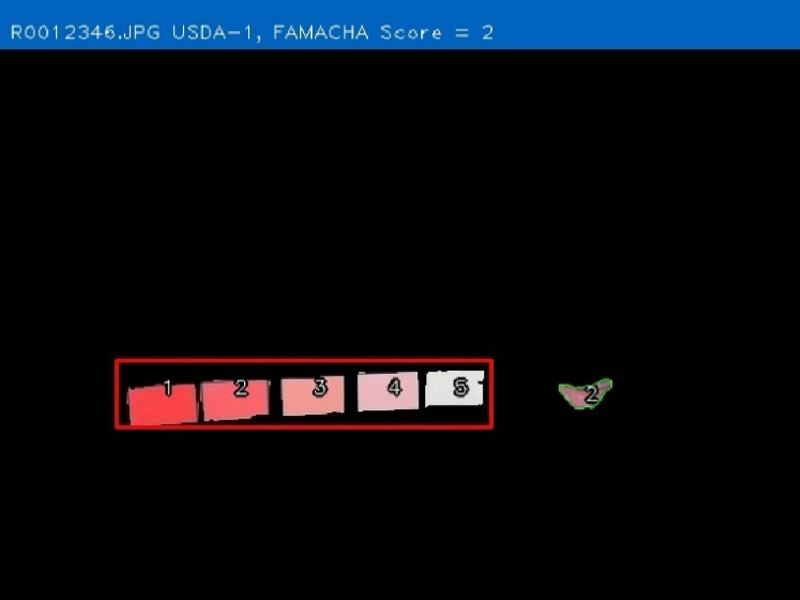

## Materials and methods

The AGIN-ICP was iteratively developed over five stages, 1) Developmental, 2) Filed Test (early), 3) Field Test (late), 4) Field Test (advanced), and 5) Controlled Test. Issues were reviewed while implementing the protocol iterations at each stage. The solutions developed were applied and tested in each subsequent developmental stage or protocol iteration. Images collected at each stage were carefully reviewed for any protocol procedure, instruction, or supporting documentation that could impact the quality of images for subsequent image analysis, and the protocol modified as needed.

Initial testing and refinement of the original protocol, which included tissue collection for DNA analyses, was conducted on goat farms in the US. Early field testing then followed in Ethiopia and Kenya. Multiple African AGIN research teams subsequently tested iterations of the AGIN-ICP, including taking the photos at the time of blood, tissue, or hair collection for DNA extraction, genotyping, and DNA sequencing; manual phenotype measures of body size; global position system (GPS) data; and demographic data including breed and birth date. Sampling teams sponsored by the FAO and Sudan joined the AGIN for the advanced field-testing phase, Stage 4. Ongoing review of field sample images to assess their quality for image analysis, led to iterative changes in the protocol (original, prototype, modified, and final versions) to improve and enhance the collection method for optimal sampling efficiency and quality of images for data extraction. Finally, images without tissue samples were collected under highly controlled conditions in the final developmental stage from US goats. These images were used to develop the PE-ISA (Woodward-Greene et al., 2022) and the software to extract animal measurements directly from the images as a proof of concept.

### Original protocol

The original protocol was named the AdaptMap Digital Phenotype Collection Method. It included the design and fabrication of calibration signs and a harness to be included in the images. Three calibration signs with a black outline for easy detection in RGB (red, green, blue) images, and color blocks of “pure” red, green, and blue, were fabricated of sturdy, light weight metal. They were designed for use with dry-erase pens to easily record sample data for each animal and capture that information directly in the images. Sample ID was recorded on all signs. Additional information recorded on the large sign only, included sex, birth date, owner, breed, sample date, location, country, camera distance from goat, and camera height. The larger sign was affixed to a harness and placed on the back of the animal for the third photograph in the series (sign view). The sign view (right side view) employs the large sign harness placed on the back of the animal. The large sign must be positioned above the underline of the belly, below the topline of the goat, and it must not obscure the joints of the front or rear legs. Finally, it must be placed perpendicular to the ground on the right side to avoid being skewed by a potentially protruding rumen. The large sign will thus be in the same plane as the goat, providing a higher quality calibration than the small sign alone. For small goats, it may be too large to place correctly, so the handler can hold it in the plane of the goat’s right side. Initially, two smaller signs were made for the goat and the handler to wear around their necks as sample identifiers in each image with the large sign only visible in the sign view. Each of these signs, and how they are meant to be used in the AGIN-ICP are shown in Figure 1.

**FIGURE 2:**
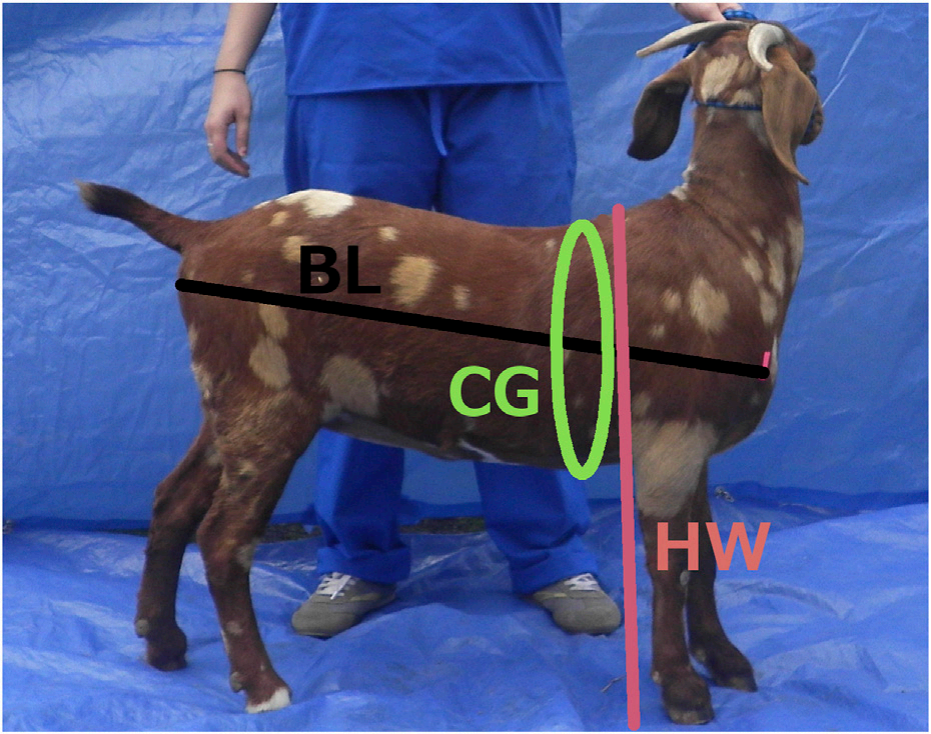
Manual (traditional) body measures taken during AGIN-ICP development.

Demographic data collected on the large sign for the prototype method was also recorded on paper, and direct physical body measures were recorded for validation of the photo measures. Physical measures included chest (heart) girth (CG), which is the circumference of the body measured just behind the elbows and at the point of the withers (shoulder bones, scapulae, at the top of the animal) (Siddiqui et al., 2008), height at the withers (HW), which is the distance perpendicular from the ground to the top of the withers (Abegaz and Awgichew, 2009), body length (BL), which is measured from the point of shoulder in the front of the animal to the point of the pin bone (tuber ischii, point of bone next to the anus) (Hopkins et al., 1970), width of the pin bones (PB) as an indicator of potential birthing difficulty, and the width between points of shoulder bones (SB) in the front as another measure of body width. A description and illustration of the body length, height at the withers, and chest girth body measures are shown in Figure 2. Body weights were recorded as references for US samples by caged pallet (walk on) scales and, wherever possible in African countries, using small portable hanging scales and slings.

**FIGURE 3:**
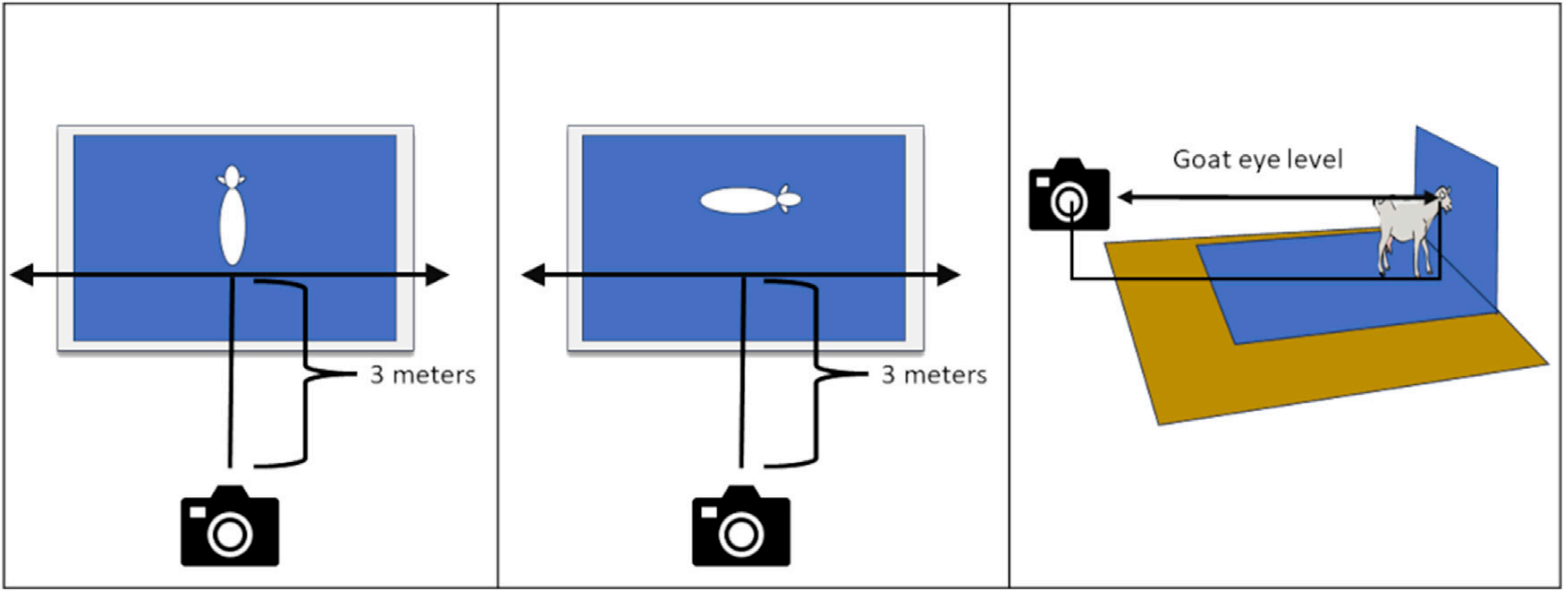
Distance and height of the camera from the goat to ensure proper perspective.

**FIGURE 4:**
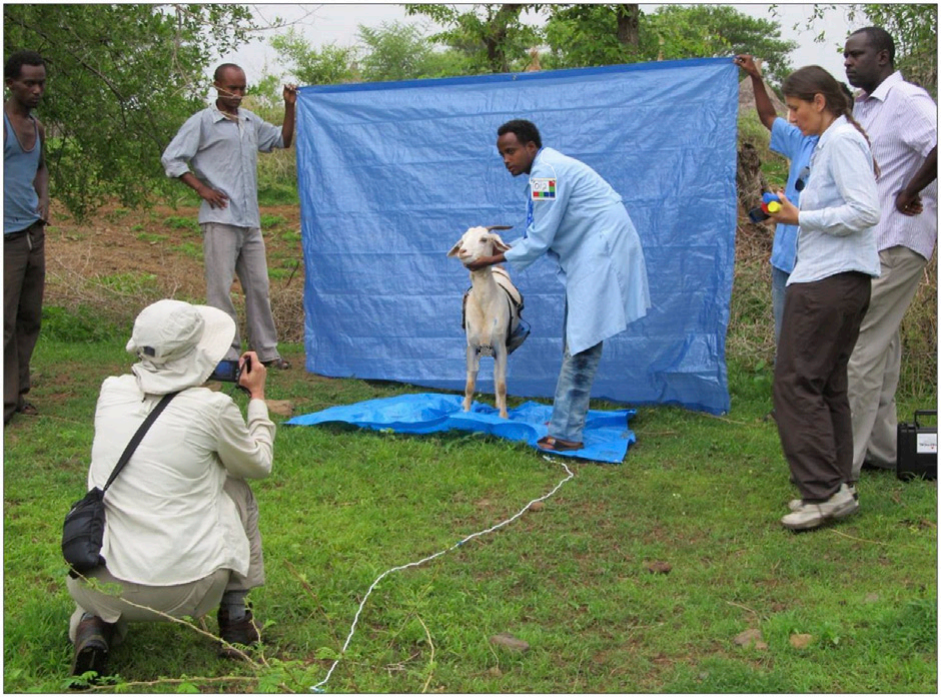
AGIN-ICP in action (Ethiopia). Note the photographer position and distance from the goat.

The poses for the image sequence were designed to minimize stress on the animal. The goat walks directly into the photo set and only makes two right one-quarter turns to achieve the body measures photos. The final two photos are for the health indicators, and are close-ups taken with the animal remaining in the final body pose (front view) position. The position of the camera and photographer is important to ensure the images have the proper perspective. This can be achieved with proper camera distance and height. The camera must be perpendicular to the goat and not closer than 3 m (10 feet). The camera height must be at the level of the goat’s eye as shown in Figure 3. A simple 3-m (10-foot) calibration rope to place on the ground between the goat and the photographer serves as a visual reminder for the photographer to identify the correct distance, and ensures the distance is maintained throughout sampling. To achieve goat eye level, the photographer must crouch or bend down (Figure 4). Alternatively they may use a tripod or sit on a small camp or milking stool. If a stool is desired, the milking stool is recommended as it can be fastened to the photographer’s body for maximum mobility, and both hands can be on the camera during sampling.

Cameras may vary, but the production of RGB images with 1,600 × 2000 resolution is preferred if possible. Higher resolution will provide greater quality images for analysis, but will require more space for storage and transfer, which can become important when working across the globe. The camera should have global position system (GPS) or global information system (GIS) capability to capture longitude, latitude, and altitude. This will enable geographic analysis, which would be valuable in assessing impacts of adaption, politico-/socioeconomics, or climate in sample populations. Cameras incorporated into mobile devices, such as smart phones or tablets would likely be adequate. Cameras used to develop the AGIN-ICP included Android based cell phones and RGB digital cameras from manufacturers such as Canon, Sony, and Ricoh. Other comparable manufacturers and systems would also work. The device or camera model and settings should be recorded. However, this data is often automatically included in the metadata of each digital image, along with GPS/GIS data, and day and time stamps.

## Results

**FIGURE 5:**
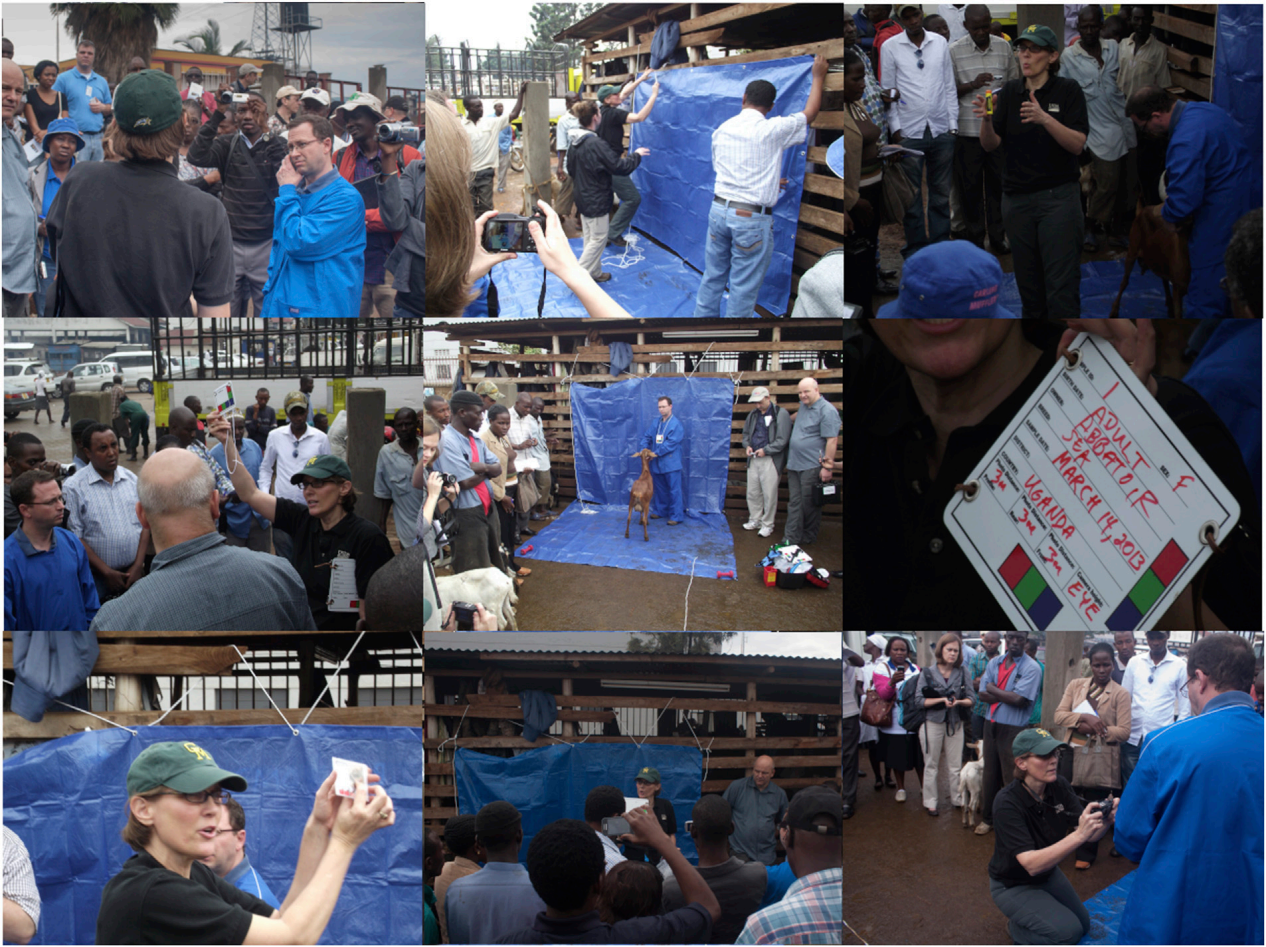
AGIN-ICP demo at AGIN II in Uganda.

**FIGURE 6:**
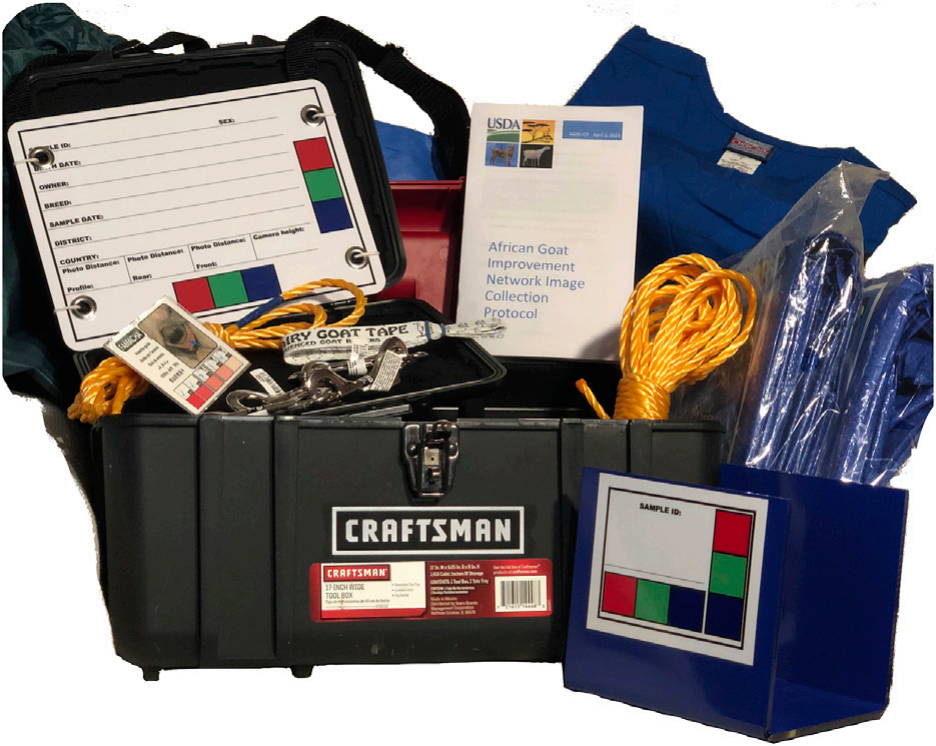
AGIN-ICP equipment kit with newly designed stationary small sign (lower right).

Results for each of 5 AGIN-ICP developmental stages (Stage 1: developmental, Stage 2: early field test, Stage 3: late field test, Stage 4: advanced field test, and Stage 5: controlled test) are summarized in Table 3. The summary includes the leader and test locations for each stage, and the changes and reasons for the changes resulting from testing in each stage. Field Testing sampling teams for Stage 3 were given a demo of AGIN-ICP at the AGIN II Meeting in Uganda as part of the field visit activities for all participants (Figure 5). Training and one-on-one discussions were also done with Stage 4 field sampling teams at the AGIN III Meeting in Ethiopia in advance of Stage 4. In Stage 3, communications were done through CWM, while in Stage 4, with leader PB coordinating, direct communications with sampling teams in the form of training and documentation, and ongoing emails and phone calls were predominant. The final AGIN-ICP is included as a supplement, as well as the Quick Start Guide developed out of Stage 3. This guide was provided to sampling teams in English and French in Stage 4. The AGIN-ICP sampling kit is pictured in Figure 6.

**TABLE 3:** Complete results summary for the African Goat Improvement Network Image Collection Protocol (AGIN-ICP) developmental stages.

Developmental Stage^a^ ^,^ ^b^	Protocol iteration	Test location	Iterative protocol changes	Reasons for changes
1. Developmental	Original	United States	1. Add blue backdrop and stand	1. Blue backdrop and stand to increase color differential of goat to the background
Lead: MJW^c^, CM^a^			2. Add 10 foot, or 3-m calibration rope	2. Ten-foot rope ensuring proper camera distance
			3. Timing sequence of demographic, tissue, and image collection	3. Timing to reduce difficulty in sampling, and inconvenience to farmers, animal handlers
2. Field Test (early)	Prototype	Ethiopia, Kenya	1. Add blue drop cloth (ground cloth)	1. Blue drop cloth to increase color differential of goat legs to the background
Lead: MJW, DM^b^			2. Affix blue backdrop to vehicle, fence, barnetc.	2. Back drop stand is heavy, and inconvenient
			3. Drop small sign on animal’s neck	3. Neck sign only on handler, due to small goats
			4. Add the ‘naked’ or ‘side’ pose	4. To provide an unobscured side view (without the sign) to extract coat color and pattern
			5. Change crayon markers for identifying the pin bones (rear pose) and points of shoulder (front pose) to bright duct tape	5. Bright duct tape to increase visibility of the marks in the images for isolation, and due to melting of crayons in the heat
3. Field Test (late)	Prototype	Uganda, Malawi, Tanzania, Mozambique, Zimbabwe	1. Interactions with multiple field sampling teams showed common questions, confusion, or field issues	1. This stage clarified the need for enhanced protocol documentation, and on accounting for field sampling conditions impacting image quality
Lead: CWM^d^			2. Iterative image review saw issues not apparent to field sampling teams, i.e., site selection, the need to avoid ‘goat like’ objects (large rocks, other equipment), cleaning the drop cloth to maintain the blue coloretc.	2. Improving field sampling team’s understanding of image processing would improve protocol implementation, leading to the development of the Quick Start Guide showing a high-quality example of each pose - connected to the phenotypic measurement to be extracted from it
			3. AGIN-ICP demo at AGIN II meeting in Uganda (ref AGIN paper)	3. Visualize method and equipment; and a question-and-answer opportunity
4. Field Test (advanced)	Modified	Burundi, Egypt, Mali, Madagascar, Tanzania, Sudan	1. Quick Start Guide produced in English and French	1. Quick Start Guide was designed to accompany the protocol, a one-page (front and back) graphical summary of the full protocol
Lead: PB^e^			2. AGIN-ICP update and informal training at the AGIN III meeting in Ethiopia (ref AGIN paper)	2, 3. Opportunity for field sampling teams in this stage to ask questions directly, examine sampling kit equipment. This connection to the lead protocol developer provided a personal connection, and a comfort level to contact her for ongoing support
			3. Ongoing support for field sampling teams was provided by email, or phone call as needed	
5. Controlled Test	Modified	United States	1. Drop the marking of pin bones (rear pose) and points of shoulder (front pose) with either crayons or tape	1, 2. Image processing confirmed little value from the front pose, pin bones, or point of shoulder
Lead: MJW			2. Drop the front pose	3. Highly controlled collection, with resulting images used to develop the PreciseEdge Image Segmentation algorithm (PE) to extract digital body measurements directly from AGIN-ICP images. This showed AGIN-ICP image measures are highly correlated to real-world animal measurements (Woodward-Greene et al., 2022). The PE algorithm is integrated into user software to return AGIN-ICP digital phenotypic measures in csv, xlsx, and xml, and labeled images for use in machine learning training set data (*manuscript in process*) for modeling more sophisticated and automated digital phenotype extraction tools
			3. Images collected in this stage were collected in a highly controlled manner, and used to develop and design the image segmentation algorithm and software to accompany the AGIN-ICP for extracting digital phenotypes from the images	

^a^ C. Mukasa led preliminary tests in Uganda, Nigeria.

^b^ D.M. led a team in Kenya.

^c^ M.J. Woodward-Greene.

^d^ C.W. Masiga.

^e^ P. Boettcher.

## Discussion

### AGIN-ICP is fit for purpose—Simple to perform, and digital extractions validated as accurate

The main objective of the AGIN-ICP sample collection is to enable reliable isolation of goats in the images collected for analysis using digital image software. The image analysis strategy involves isolating the region of interest (ROI) containing the goat or the sign in the image, creating an image mask, and calibrating the pixel values for size and color using image feature detection techniques. Stage 5 was designed to collect images under precise conditions using the most mature iteration of the protocol. These images were used to validate that the AGIN-ICP could meet this objective.

Regarding the overall importance of the many detailed procedures in the final AGIN-ICP, they meet the stated objective of being simple to implement. However, this simplicity may belie their critical importance. It is crucial to follow these steps precisely to obtain the highest quality images for digital phenotype extraction. Stage 3 revealed many subtle variations in interpretation of the protocol tasks by different sampling teams, and the negative impact these variations had on image quality. This made it more difficult, and in some cases impossible to obtain digital measurements. The lack of full understanding of the purpose of each step led to poor site selection, and the failure to understand the need to keep the tarps clean, and thus blue in color, led to reduced contrast of the blue background, and reduced image quality for data extraction.

Considering the time, expense, and materials devoted to field sample collections, combined with the importance of this type of data collection, sampling teams must have a solid understanding of the protocol steps and their purpose for image data extraction. Ultimately, while performing the AGIN-ICP correctly under field conditions is not difficult, it is also very easy to get it wrong if attention to detail is lacking. To address this issue, explanatory images were added to the AGIN-ICP to demonstrate problematic images and how to correct them in the field. Additionally, the Quick Start Guide clearly explained the purpose for each pose. Moving forward, examples of output images may prove extremely helpful, but these were not available during the AGIN-ICP development as the software was created using the images taken in Stage 5.

Companion software to process and extract digital phenotypes from AGIN-ICP images was created for this critical step in the development of AGIN-ICP process and for practical application (*manuscript in process*) of AGIN-ICP. The PE-ISA was developed and embedded in the software to find and then isolate the ROI (i.e., remove all background from image) and produce an intermediate labeled image of the ROI. The supporting software was designed to take the intermediate labeled image from the PE-ISA as input. The software automatically measures and calibrates the ROI in AGIN-ICP images, and seamlessly returns digital data to users in Excel (xlsx), comma separated (csv), or extensible markup language (xml) formats, as well as providing the intermediate, and final labeled images for review or presentation (see the final labeled output images on the right column in Tables 1, 2).

The PE-ISA and the associated software developed, allowed us to validate that the AGIN-ICP does in fact, deliver images that can provide data for precise digital phenotypic measurements from the images; and that the extracted digital measurements are highly correlated with real-world (traditional) livestock measurements. Manual versus digital extracted body measurements Pearson correlation coefficients for height, length, and girth measures were 0.931, 0.943, and 0.893, respectively (Woodward-Greene et al., 2022). These extracted phenotypic values may in turn be further analyzed to return a body weight prediction, coat color, coat pattern, or other values. The output labeled images and data files describing the labels could be used for machine learning training and test sets to develop models for automated prediction, decision, or image processing tools.

### The AGIN collaboration platform addresses multi-national collaboration challenges

Challenges encountered in implementing and developing the AGIN-ICP internationally included differences in time, distance, and language. The overall coordination and organization of the AGIN, provided resources, collaborators, visibility, and support from AGIN, as well as support from AGIN organizational members such as the USDA and the FAO. In combination with the AdaptMap project (Stella et al., 2018), resources, and expertise from across a broad spectrum of biological, social, and political domain experts, were readily available to address technical and logistical challenges in Africa, and formed solid and lasting relationships. The AGIN provided opportunities for students, mid and senior level researchers, farmers, and government and local officials from many countries to interact as equals, facilitating a free flow of information and exchange of ideas that became a hallmark of AGIN, and key to many of its successes. In this example of the image collection protocol development, the technical vision for AGIN-ICP was grounded in these broad perspectives, ensuring a protocol that was practical, while also delivering the technical requirements to provide accurate phenotypes from digital images.

Each iteration of the AGIN-ICP process included improved instructions for collecting demographic data, taking manual body measurements, and collection and storage of DNA samples (blood, tissue, or hair). Sampling team leaders communicated predominantly by email to clarify how to implement all aspects of the protocol. Together with these ongoing enhancements to AGIN-ICP and equipment, methods for communication and training on best practices for optimal implementation of the AGIN-ICP were steadily improving as well. These enhancements included the creation of a Quick Start Guide in English and French on a single page, and a graphic of the Digital Analysis Workflow on the reverse of that page. The Quick Start Guide was applied in Stage 4 and included images to demonstrate the poses used in the AGIN-ICP along with brief explanatory captions. The Digital Image Analysis Workflow is a diagram explaining the purpose of each pose, i.e., what digital phenotype(s) are extracted from each image pose. Development and evolution of the sampling kit with everything needed except the camera or cell phone camera, was also an iterative and collaborative process, with a kit provided to each sampling team, shown in Figure 6. A listing of the photo sampling kit contents is found on the next to last page of the AGIN-ICP. The AGIN-ICP, the Quick Start Guides with the Digital Image Analysis Workflow, and the Hair Collection Procedure adapted for AGIN-ICP are included in the Supplementary Material.

### Moving the goat

The goat to be sampled requires minimal preparation. Demographic information is collected for each sample as described in the section on calibration signs. Goats are led into the photo shoot area by hand, neck chain, or halter. Animal identification may include ear tags, tattoos, or farmer memory. Generally, sampling teams did not have difficulty working with the animals to apply the AGIN-ICP; however, the FAMACHA and the tooth poses were the most difficult, as they required a higher degree of human—animal interaction compared to the rear, side, and sign, or ‘body measures’ poses. For the body measures photos, the disposition of the goats was judged to be overwhelmingly cooperative in all Stages of AGIN-ICP development, with few exceptions.

### Stage 1 developmental in the US—Order of operations and site set-up

**FIGURE 7:**
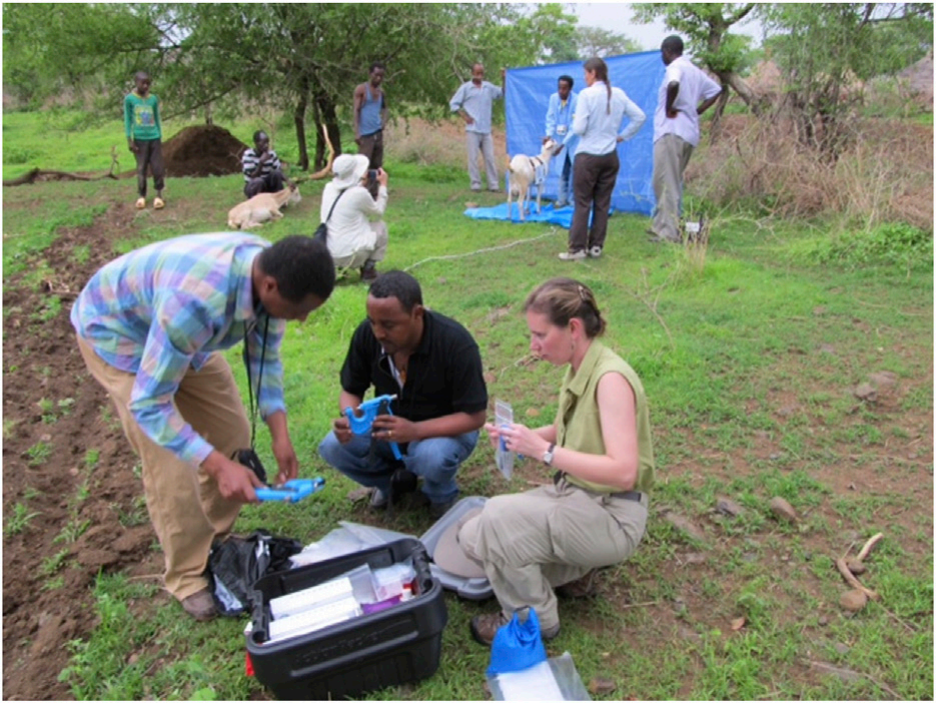
Ethiopia, HJH, SA, TG prepare for manual body measurements and DNA collection as the first goat is photographed by MJW, AH, B. Rischkowsky and local farmers assist.

Stage 1 developmental testing for the original protocol was led by MJW and conducted in the US. This stage confirmed the efficiency of the method in conjunction with tissue sampling for DNA. Two teams working concurrently, but the tasks were staggered, as the tasks of each team took the same amount of time to complete. One team consisted of the photographer (MJW) and a goat handler. This team was responsible for recording demographics, marking pin bones and shoulders, and taking photos. The second team (HJH) recorded body measures and collected DNA. The timing of the two teams, and the order of operations was determined to minimize animal stress and maximize overall efficiency. The first sampling (photograph) team would record the demographic data, mark the pin bones and shoulders, and take the photo series. Next, that same animal was moved to the second team for body measures recording, with DNA collected last, as it may involve stressful tissue (ear punch) sampling, hair pulling, or needle insertion for a blood draw. While the first animal moved on to the second team, the next goat would begin sampling by the photography team, and thus two goats were sampled simultaneously. The full sample group would start with a single goat recorded by the photography team, and end with another single goat with the DNA and body measuring team. Two goats were being sampled concurrently at all other times (Figure 7). Review of Stage 1 images demonstrated that isolating the goats in the images would be difficult because of the similarity of the goat, background, and handler clothing and skin colors. Thus, a blue backdrop was added to the protocol to aid in image analysis. These issues were further addressed in Stage 2.

### Stage 2 field testing (early) Ethiopia and Kenya—Site set-up, equipment, add the side pose

Stage 2 began with MJW, HJH, JS, AH, BAR, SA, TG, and other AGIN participants working with local farmers and leadership to conduct field sampling in Ethiopia, followed by MJW and HJH sampling with DM, AbK and other AGIN participants and communities in Kenya. Stage 2 focused on method modification and refinement of the equipment needed. The Stage 1 had confirmed the efficacy of the order of operations and procedures discussed above, and included the addition of a blue backdrop, and a blue lab coat for the handler to enhance image analysis isolation of the region containing goat. In general terms, the isolation of the goat in the image at this stage, and thus iterations from this point relied on three main image features integral to the image collection method,

• The goat, surrounded by a blue background, can be more easily isolated in image analysis because the goat does not have any blue regions.
• The goat has a limited range of size, and
• The goat and the tarp are in the center of the image.

A portable, free-standing device was constructed in the US and transported to Ethiopia and Kenya to hang the blue backdrop added in Stage 1. The kit for the stand included tripods, ropes, blue tarps, and weights to keep tarps from blowing away. While portable, at roughly 200 pounds, the portable stand kit was burdensome, and was quickly tossed aside. A photograph of it is included in the AGIN-ICP if anyone would see a need to use such a tool. The AGIN-ICP and kit provides a simple rope for more convenient tying of the blue backdrop to buildings, trees, or fences, etc. Ultimately, it was found in Stage 2 that the vehicle transporting the sampling teams and equipment often proved to be a reliable place to affix the backdrop in a variety of situations, thus the stand was not included in the sampling kit provided to subsequent teams. This simplification enhanced the convenience, and reduced costs, and minimized the size and weight of the overall AGIN-ICP equipment kit.

After testing the backdrop on the first sampling group in Ethiopia, daily review of the images showed that a blue floor drop cloth would also be needed, as the lower portion of the goat would blend in with the soil and dust. Extra tarps were available and employed immediately for the remaining Stage 2 Ethiopian and Kenyan samples. Similarly, it was discovered from the images that the blue lab coat was effective, however, the legs, trousers or skirt of the handlers may still blend in with the goat in some situations, potentially interfering with isolating the goat in image analysis. It was determined while sampling in Ethiopia and Kenya for Stage 2, that blue surgical scrubs (shirt and pants or skirt) would provide more complete coverage. However, these items were not available, and thus the blue scrubs were not employed until subsequent field sampling by collaborators in Stage 3.

The small sign meant to hang from the goat’s neck for calibration did not work well, especially in African goats when comparing to Stage 1 in the US. The African goats were generally smaller than the US goats. The small sign hanging on the goat’s neck obscured most of the front of the goat’s body. The small sign continued to be worn around the handler’s neck, to ensure the sample identification number was in all the photos. Finally, the large calibration sign was fastened into a harness to be laid over the goat’s back, with the sign on the right side. A counterweight on the left side of the harness was added to keep it in place. It lies on the back of the goat, like a saddle, so the sign can be adjusted to the correct position with the top of the sign below the backbone and bottom of the sign above the bottom of the belly, for the sign pose image. Also because of the smaller size of the African goats compared to the US goats originally tested, the inside of the large sign harness, which was black in color, would sometimes be visible hanging down on the opposite (left) side of the goat below the belly, which could interfere with isolating the goat in image analysis. Thus, the harness was modified to have the backside of the harness completely covered in blue tape to eliminate this noise from the image analysis. The signs and their placement are shown in Figure 1.

Finally, it became clear that the sign view pose of the animal with the calibration harness in place, covers up distinctive patterns or colors in the coat which are of interest in phenome and genome research. Thus, a sixth photo of the right side without the large calibration sign, named the ‘naked’ or ‘side’ view, was added to the protocol starting with the Stage 2 Kenyan samples. This addition made a total of four poses to obtain body measures, coat color and coat pattern, plus the two close-up images used for the teeth age and soundness, and the FAMACHA score.

### Stage 3 field testing (late) in 5 African countries—Identify communication, training, support needs

Stage 3 involved six sampling teams in Uganda, Malawi, Tanzania, Mozambique, and Zimbabwe implementing the prototype iteration of AGIN-ICP. The blue scrubs were included in the kit for all but the first team. The focus continued to be method modification and equipment refinement, with a strong communications and training development element added. The Stage 3 lead, CWM, was able to meet with MJW and HJH in Kenya before their sampling for a one-on-one review and discussion of the procedures and equipment and to discuss how the field testing was going in Stage 2. CWM picked up the first sampling kit there, and so did not have the blue scrubs as subsequent Stage 3 teams did.

Communication with Stage 3 sampling teams were through the Stage 3 lead, CWM, and done by email with an occasional phone or Skype (video-telecommunication) call. In person training, including DNA sampling occurred when the AGIN-ICP prototype was demonstrated at a Ugandan livestock market as part of the second conference and workshop for AGIN members at AGIN II (Figure 5). This demonstration included the blue back and floor tarps, the blue scrubs, the small sign worn by the handler, and the added naked view for a total of 6 images per goat. Discussions and presentations of the protocol Field Testing progress were also shared at the AGIN III workshop in Ethiopia. Stage 3 offered much in terms of understanding needed improvements in communications, in particular clarity in the protocol instructions and support documentation for field sampling teams. Ongoing assessment of field images being collected revealed gaps in the instructions, and several training handouts were developed.

**FIGURE 8:**
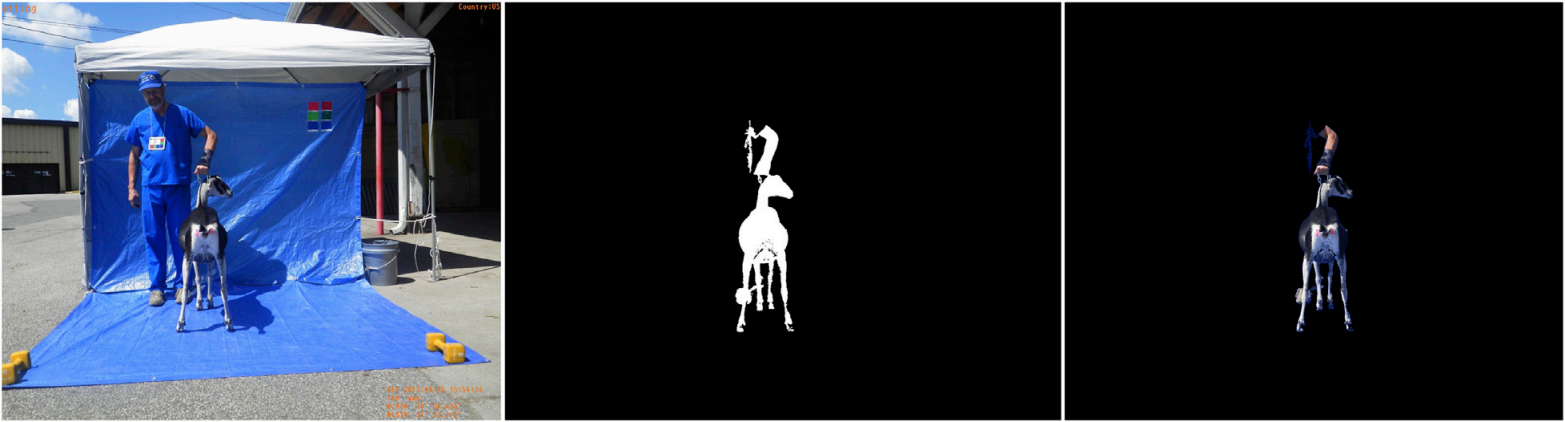
Early mask generation and implementation showing effectiveness of blue tarp and clothing, and importance of handler standing away from the goat to remove non-goat features (human arms, shoes). Testing of the images revealed any variety of human skin and goat colors are over 90% similar.

Stage 3 ongoing images review also revealed the importance of the handler posing with the goat correctly, i.e., standing away from the goat especially if the handler is unable to wear the blue clothing. Figure 8 shows early attempts to isolate the goat using color feature detection. Note that any part of the handler not covered in blue can interfere with the isolation of the goat from the background. The final image analysis software has been improved, and is robust to handle such noise due to varying image quality in terms of goat pose, handler position, lighting, or camera settings. However, an important part of an efficient solution is to communicate clearly that the highest quality images are produced when there is nothing between the goat and the blue background. This problem is solved completely by the handler taking one or two steps away from the goat’s body if possible; and if this can be done, the blue scrubs may not be necessary. Sampling goats can be tiring work, and maintaining precision in collecting the images per the AGIN-ICP a challenge. The steps again are easy to do, but also easy to forget as the sampling day wears on. If the handler would do both, that is to wear all blue, and step away from the body of each goat, this would provide redundancy to ensure nothing except the goat is in the center of all the images.

Equally important and impacting the image analysis is site selection and set-up. Stage 3 images were sometimes taken in less-than-ideal conditions. For example, the photography site should be free of unnecessary, large, or goat sized objects. The location should be as level as possible. The light source should be behind the photographer. Example images of poor vs. high quality collection site, set-ups, and execution of the photos, including FAMACHA images, were compiled and shared with sampling teams, and added to the AGIN-ICP, as shown in Tables 1, 2 of the protocol (Supplementary Material). This graphical communications approach was helpful, as it was not as dependent on trainer and trainee speaking the same language.

Additionally, photographers were strongly encouraged to take practice photos and return them to MJW for review and comment prior to going out on a sampling tour. This interactive approach was effective; however, it was still difficult to anticipate all the varied questions and situations that would come about under field conditions. Stage 3 sampling teams sometimes had difficulty troubleshooting unexpected sampling site or equipment issues to the extent needed to maintain image quality for analyses. To provide an example, one sampling team set up the blue ground cloth tarp on uneven ground that was littered with large rocks, despite having a nearly level surface available, as was seen in the images taken that day. The uneven site was selected so that the backdrop could be easily hung from a building, rather than giving preference to level ground. The resulting images had the goats correctly positioned with all blue in the background, but they were standing behind the rocks, which obscured their feet and legs, making automated extraction of the goat body height from these images impossible. This challenge in trouble shooting the site selection stemmed from a lack of full understanding of how each image pose would ultimately be analyzed, and it inspired the development of the AdaptMap Quick Start Guide, and the Digital Image Analysis Workflow graphic (Supplementary Material).

### Stage 4 field testing (advanced) 6 African countries–communication, training, support needs

The Quick Start Guide was provided to sampling teams in Stage 4. Additionally in Stage 4, a face-to-face update, training, and discussions of the modified protocol iteration, and image analysis was completed as part of the third AGIN workshop in Ethiopia. This direct training and informal discussions, along with the protocol modifications and the Quick Start Guide, proved the most effective training and communication approaches to date to obtain high quality images. Specific protocol and equipment modifications coming out of Stages 2 and 3 and implemented in Stage 4, included the need to increase visibility of the pin bone and shoulder bone markings, and to address issues in using the small sign on the handler’s neck, which was often skewed, not fully visible, or omitted altogether. The visibility of the livestock crayon markings on the pin bones and shoulder bones in the Stage 2 and 3 images was also not adequate. In addition, some sampling teams had the crayons melt in the heat, rendering them useless for any remaining samples.

**FIGURE 9:**
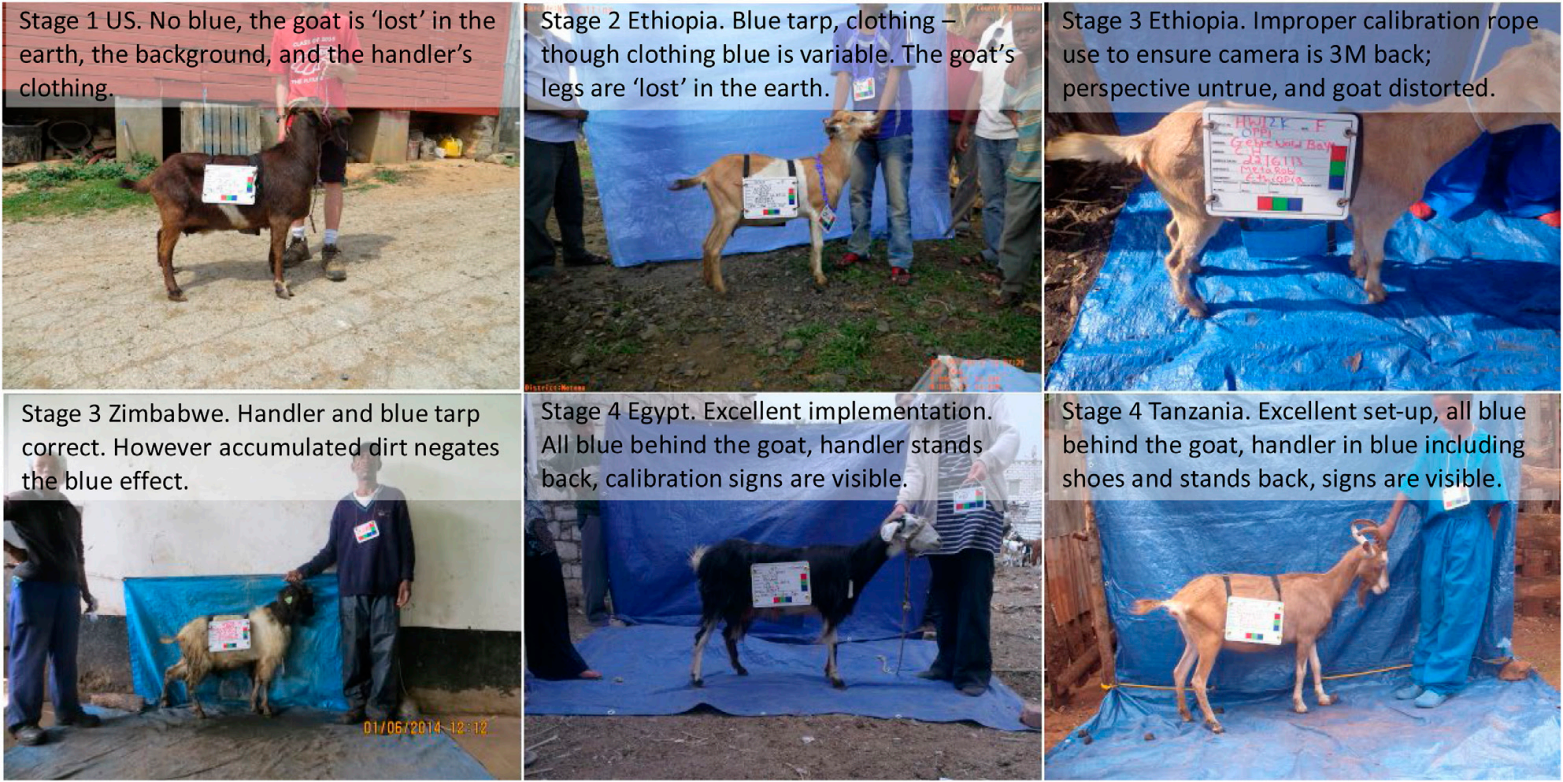
Modification examples and impact on implementation quality through developmental stages.

To address the issues with the livestock crayons, a method was devised during Stage 4, on how to prepare duct tape strips to mark the pin bones and points of shoulder bones instead of crayons. A few field sampling teams in Stage 4 were provided these instructions, and bright pink duct tape. The continual review of the images collected by sampling teams showed that despite communication efforts, many sampling teams were unable to properly locate or mark the pin or shoulder bones with either duct tape or livestock crayons. This problem created inconsistency in the digital measurements like what had been seen in manual body measures. These inconsistencies were precisely what the AGIN-ICP aimed to overcome. The issues addressed in the protocol iterations up to this Stage, i.e., the effects of the addition of blue tarps and clothing, and improving communication on site selection, and proper or optimal use of the equipment and other procedures over the developmental stages are shown in Figure 9.

### Stage 5 controlled testing US final collection of images to develop image phenotype extraction

**FIGURE 10:**
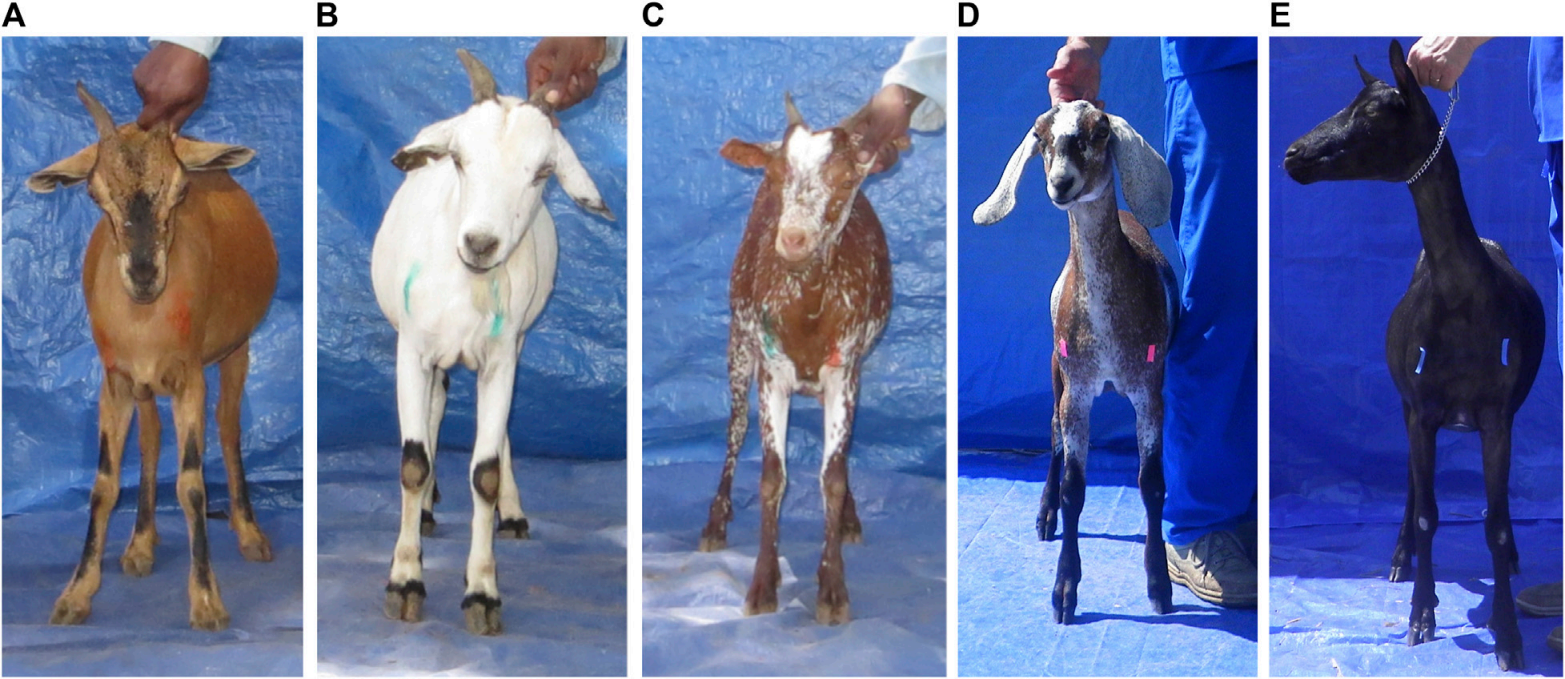
Kenya Stage 2 bone marking with livestock crayons **(A–C)**, and United States Stage 5 testing of duct (pink) and painter’s (blue) tape **(D,E)**. Both tapes were more visible, but painter’s tape did not stay on the goat. Bone marking was deemed ineffective for properly locating bones, and the markings were also difficult to pick up in image analysis. It was dropped from the AGIN-ICP.

Stage 5 occurred concurrently with Stage 4, and allowed for careful, highly controlled testing of new or modified procedures and equipment as the final African sampling progressed. The addition of the sixth pose image, the ‘naked’ or ‘side’ view to extract coat color and pattern, was fully implemented in Stage 5. The modifications to the AGIN-ICP tested were a direct result of issues revealed in Field Testing Stages 2, 3, and 4, and included low visibility of pin bone and shoulder bone markings. Additionally, Stage 5 included testing of multiple cameras and light levels. To address the pin bone and shoulder bone marking, blue painter’s tape, and bright pink duct tape were tested as possible alternatives to the livestock crayons. The blue tape was not sticky enough to stay in place through the photo series, and did not work with the approach to eliminate the blue background with the software. The pink duct tape proved to be far superior in visibility compared to the crayons, and it stayed in place on the animals better as compared to the blue painter’s tape (Figure 10).

To facilitate using the duct tape, a procedure was added to the prototype protocol, describing how to prepare small strips of tape in advance by scoring deep into the duct tape roll with a razor at 1-cm intervals, and this procedure was shared with some of the Stage 4 sampling teams. This allowed easy and quick access to the strips during sampling. Sampling teams were cautioned that the tape should be removed from the goat’s body when finished sampling, so the goats would not consume it. Despite the clear improvement seeing the markings with duct tape, persistent issues of proper placement of the marks rendered the value of this step negligible, and it was ultimately eliminated in the final AGIN-ICP. The width and volume of the animal’s body is instead determined by isolating and calibrating the rear pose using the PE-ISA developed for AGIN-ICP images. The prototype protocol used in Stage 4 and Stage 5 demonstrated a stringent, yet easily applied method that can collect high enough quality digital images under field sampling conditions, such that the subject ROI can be isolated in image analysis for extraction of phenotypic data. The final AGIN-ICP is presented in the Supplementary Material, and takes into consideration all the issues overcome, lessons learned, with advice and feedback from sampling teams, farmers, and others, especially AGIN members, who were updated at each AGIN gathering of the AGIN-ICP development progress and status.

### AGIN-ICP sustainability, future, and the legacy of the AGIN collaboration model

**FIGURE 11:**
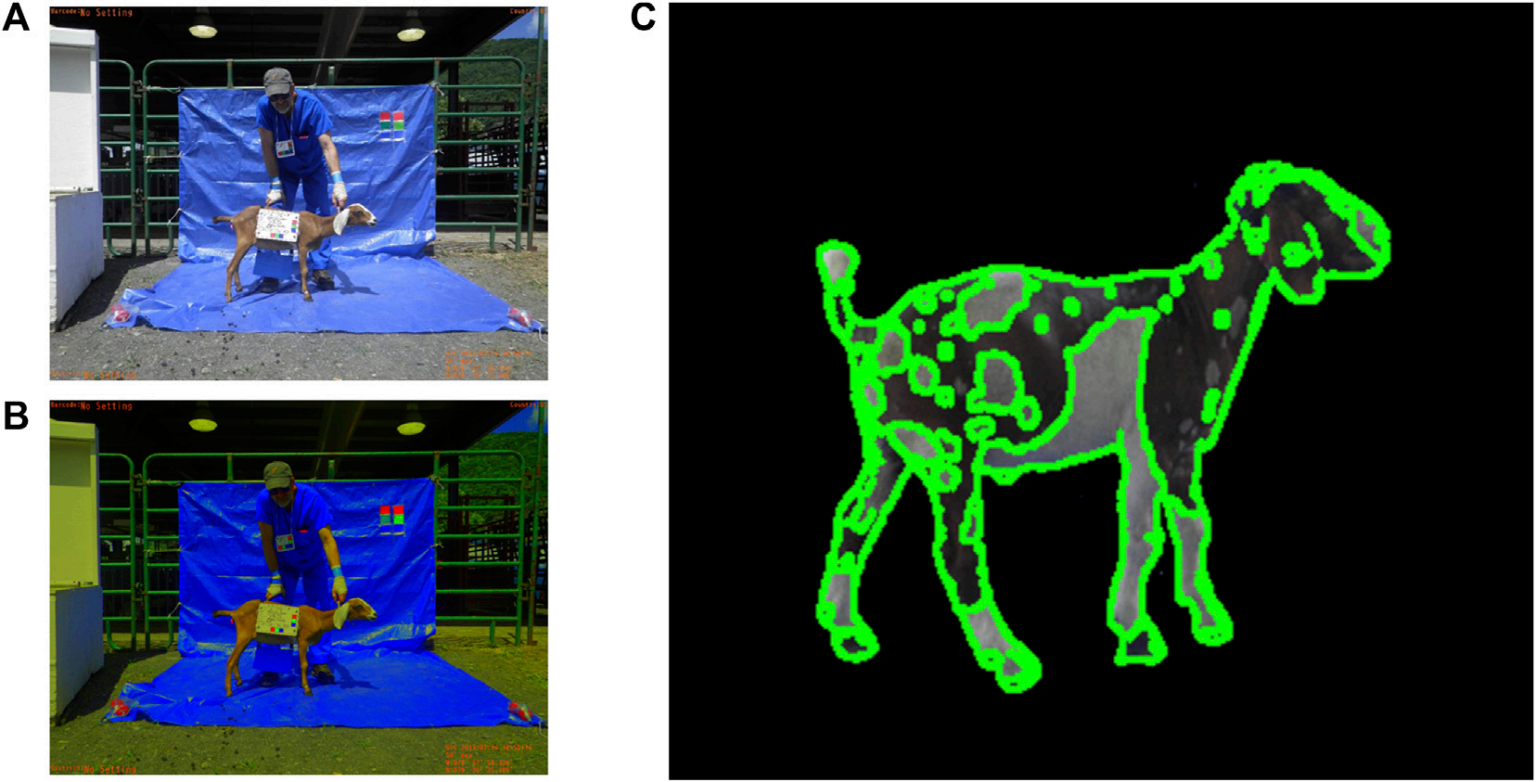
Preliminary software development for coat color calibration **(A,B)** and coat pattern **(C)** recognition.

The AGIN-ICP is just one example success story that is a direct product of the AGIN multi-national collaboration platform, and though the USAID funding has ended for AGIN, it has set a precedent for collaboration that is being repeated by other research groups. Further, the relationships and the capacity built both in terms of human resources and technical innovations ensure that the work of AGIN will be sustained, and continue to evolve (Van Tassell et al., 2023). The software development for processing AGIN-ICP images has demonstrated proof of concept for accurate digital animal body measurements of height, length, and chest girth. Software development continues with teeth and FAMACHA measurements extracted (Table 2), and validation studies for these phenotypes are planned. Work is also ongoing to calibrate coat color for a numerical value meaningful across varied samples and settings, and to extract and vectorize coat pattern (Figure 11).

**FIGURE 12:**
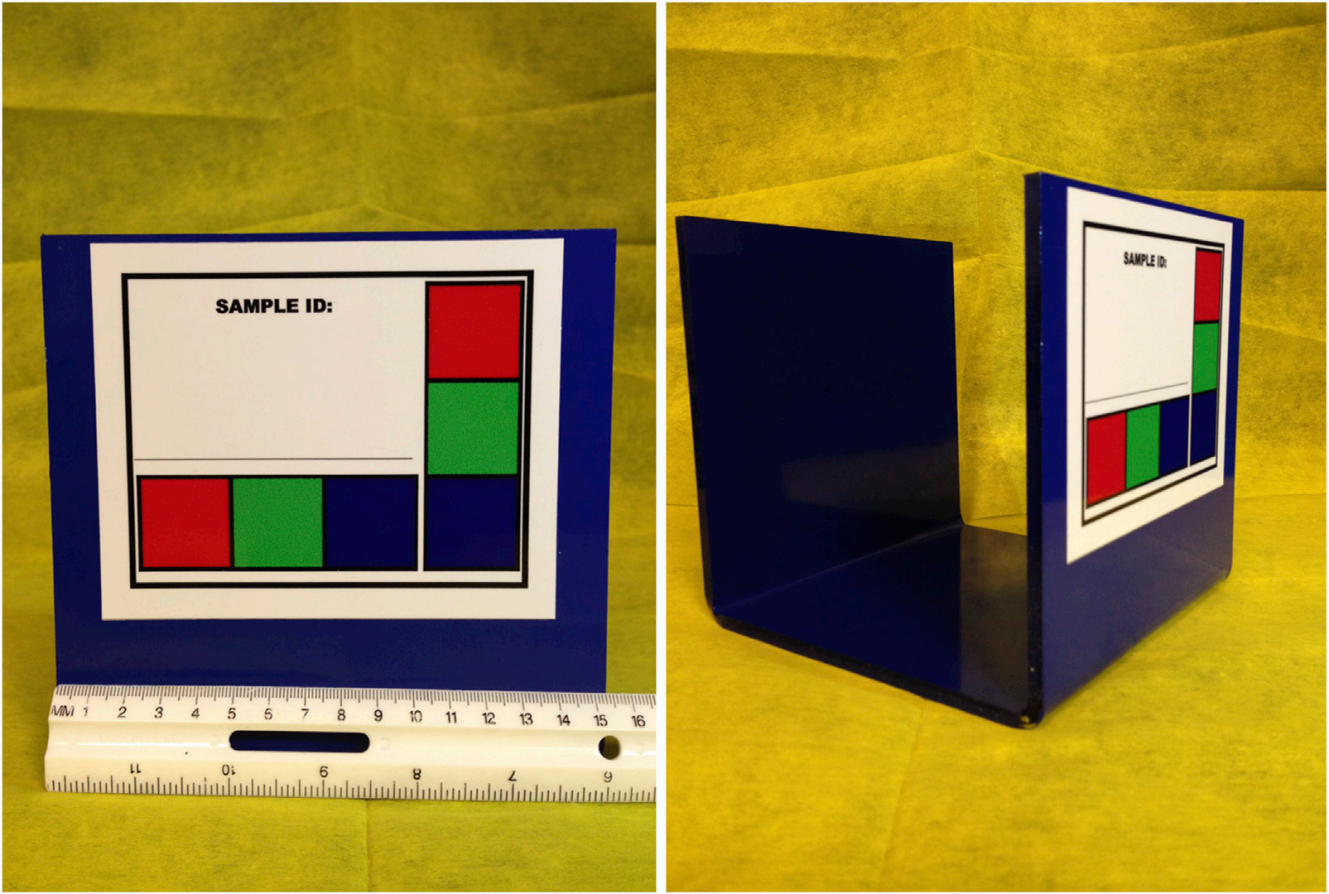
Re-fabricated small calibration sign for stand-alone use will minimize its skewing or omission in body measure photos.

As the AGIN-ICP is used in more and more sampling settings, its history of evolution is likely to continue. Going forward, MJW has redesigned some of the equipment to better address known issues or to simplify the protocol. For example, the small calibration sign that was too big for the goats, and too unstable for wearing by animal handlers, has been redesigned for standalone use, as shown in Figure 12. Extensive work extracting body measures data from AGIN-ICP images shows that only two photos (a rear view and sign view) are needed to extract accurate body measurements. Sampling teams who do not need the other measurements may eliminate the other poses in that case.

Finally, automated extraction of GPS data embedded in the images is a feature planned for a future version of the software. Record keeping and analyses such as growth rate over time, would be a helpful feature for farmers or others selecting animals for breeding, marketing, or to monitor health. These data, combined with the phenotype data could prove valuable for researchers, farmers, and veterinarians who wish to assess their stock in the context of the local climate and production systems, so they can make informed decisions based on accurate and relevant information.

## Conclusion

The African Goat Improvement Network Image Collection Protocol (AGIN-ICP) has shown that it is an efficient, easily deployable, inexpensive method to collect digital images from livestock without causing the animals, or the handlers undue stress. The resulting images collected with the AGIN-ICP were used to develop the PreciseEdge Image Segmentation Algorithm (PE-ISA), which returns digital phenotypes from AGIN-ICP collected images that are highly correlated to analogous, real-world (traditional) animal phenotype values, with Pearson correlation coefficients for height (0.931), length (0.943), and girth (0.893) (Woodward-Greene et al., 2022). Sampling teams using the AGIN-ICP must understand that despite the simplicity of the protocol, attention to detail and an understanding of the purpose for each step is critical to obtaining images that can be used to extract digital phenotypes as intended.

The phenotypic data from this process is ‘born digital’ and thus can save time, effort, and errors because data entry is not needed. The accompanying user software that embeds the PE-ISA for easy processing of images was developed using images collected by the AGIN-ICP in Stage 5. It will provide users with a variety of data formats for subsequent phenotypic analyses; as well as labeled digital images that could be used in artificial intelligence machine learning test sets, for data modeling to advance decision tool innovations (*manuscript in process*). The AGIN-ICP is a case study in the AGIN multi-level collaborative model to empower all levels of stakeholders in a CBBP. This work, and all of the authors benefitted significantly from the breadth of expertise and synergy of shared purpose fostered and supported by the AGIN structure, members, partners, and sponsors. Workers engaged in the CBBP, from farmers, to students, to junior and senior researchers, as well as local, regional, and national government officials and sponsors from multiple countries all had a significant role in the success of the AGIN-ICP.

## Data Availability

The original contributions presented in the study are included in the article/Supplementary Material, further inquiries can be directed to the corresponding author.
