# Supplementary material for: Using the community-based breeding program (CBBP) model as a collaborative platform to develop the African Goat Improvement Network—Image collection protocol (AGIN-ICP) with mobile technology for data collection and management of livestock phenotypes
Source: Front Genet. 2023 Sep 6;14:1200770. doi: 10.3389/fgene.2023.1200770 (PMC10512022; doi:10.3389/fgene.2023.1200770)
Supplement: Supplementary file 1 [file Presentation1.pdf]

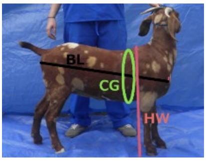

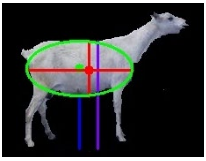

2023

African Goat Improvement Network Image Collection Protocol

AGIN-ICP

AGIN-ICP

The photographer must insist on as level a spot as possible, with plenty of space (~8x8m min) to move around. Be aware of lighting, i.e., glare or shadows that can obscure the images. It is recommended to read a primer on photography and lighting issues, especially for outdoor photography, i.e., take care not to shoot directly into the sun, etc.

1. DATA SIGNS: RECORD the following information with DRY ERASABLE markers. (Permanent markers will ruin the signs.) The SMALL sign will be included on the handler in EACH image, and the LARGE sign will be in the third, or 'sign shot' (**Figure 1**, **Figure 2**, **Figure 3**).
    a. Sample ID on smaller neck sign (**Figure 2**), for handler- must be visible in EACH photo.
    b. Larger harness sign for goat (**Figure 1**), must be visible in shot 3, 'sign shot' (**Figure 3**).
        i. Sample ID
        ii. Sex
        iii. Birth Date
        iv. Owner
        v. Breed
        vi. Sample Date
        vii. District/Location
        viii. Country
        ix. Distance of goat from camera, profile,

<table>
<tr>
<td>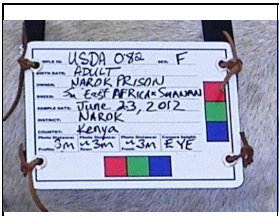
</td>
<td>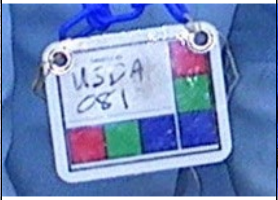
</td>
<td>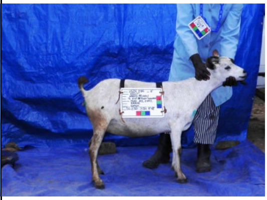</td>
</tr>
<tr>
<td>Figure 1. Data sign for harness.</td>
<td>Figure 2. Small sign for handler.</td>
<td>Figure 3. Positions for signs.</td>
</tr>
</table>

rear, and front photos

x. Camera height in line with goat eye level: if needed use a tripod or similar standard tool, or sit on a camp stool or chair so the camera angle is standard.

c. Place the small sign over the handler’s neck- BE SURE IT IS VISIBLE IN PHOTOS

d. Lay the sign/harness gently over the back of the goat as shown in **Figure 3**.

e. NOTE: Sample Identification: A matching ID number needs to be used to associate the sample (for DNA extraction if collecting biological samples, see BIOLOGICAL DNA SAMPLING) to the photo and physical body measurements. Therefore, the same ID needs to be on each of these items. Pre-printed stickers are recommended.

2. DIGITAL PHOTOS: RESOLUTION 1600x2000, spot focus on sign with sign Camera- digital, record pixel rating (model, etc.), include GIS data if available in camera settings.
    a. GIS: document GIS (GPS coordinates, elevation) if not automatic on your digital camera.
    b. Fill in the dry erase data signs; be sure they are visible in photos as described below.
    c. PHOTOS
        i. SET UP (also see table 1 and review carefully)
            1. Select a LEVEL site with sun/light at your back.
            2. Floor and back drop tarps MUST be linked together with metal snap clips provided; attach clips in REAR of tarps so they are not visible in photos.
            3. Use small weights to secure the floor tarp, so it does not interfere with isolating the goat in the final image analysis.
            4. Tarps must be as free of folds and ripples as possible- as these cause dark lines in the photos, also be aware of shadows cast on the tarps from the

front or rear, and minimize them as much as possible.

ii. POSING the animal and the handler

1. The HANDLER wears BLUE so as not to interfere with digital analysis, BLUE SCRUBS are included in the photo kit, blue gloves are highly recommended, as well as blue shoes.
2. ***The HANDLER must wear the small calibration sign, and ensure it is visible to the camera in all distance shots.***
3. Pose the goat with feet square below the body, naturally with no stress (pulling) by or on the handler if possible.
4. ***Handler must stand away from the goat as much as possible, so only the blue tarps are behind the goat in the finished images.***

iii. CAMERA position (**Figure 4**)

1. THREE METERS (10 feet) back- measuring tape or calibration rope provided.
2. Perpendicular to the goat position
3. Camera Low, at GOAT EYE LEVEL, bend down, or use a tripod (fixed height) or

camp stool for proper proportions in the image, and to minimize between photo variations.

4. Profile (FULL BODY) side (naked) and sign shots, head facing RIGHT to minimize possibly protruding rumen. HANDLER- please TRY not to obscure the lower jaw or face, so that beard, wattles, and nose/face shape are visible.

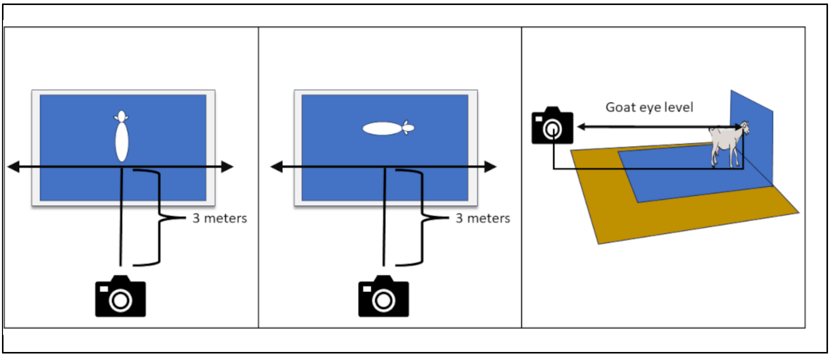

Figure 4. Camera position, 3 meters back, perpendicular to the goat, at goat-eye level.

iii. PHOTO SERIES (**Figure 6**). ***Take photos in the order shown for consistency, and in case data signs are obscured in photos, sample ID may be determined by photo series pattern.***

1. Rear shot
2. Naked shot

3. Sign shot, place large sign harness on the goat, as perpendicular as possible to the ground ***(this is the ONLY time a sign is placed on the goat)***
4. Front shot (optional)

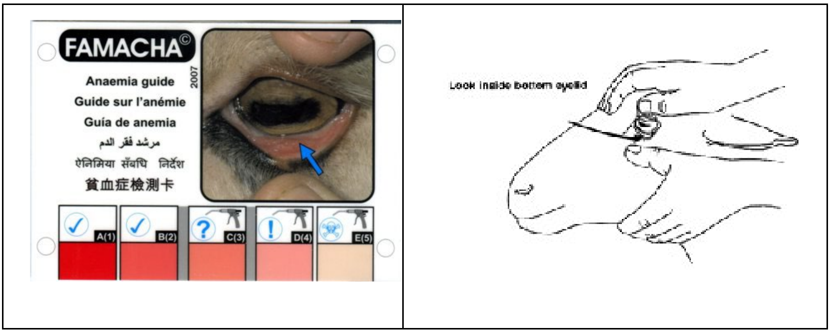

Figure 5. FAMACHA card and proper exposure of conjunctiva (lower inner eyelid).

5. Teeth shot- Pull lips apart, exposing teeth (**Figure 6**, Shot 5.)

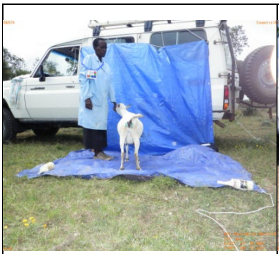	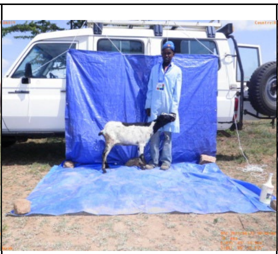	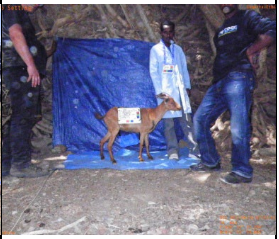
Shot 1: Rear view	Shot 2: Side/Naked view, profile	Shot 3: Sign view, profile
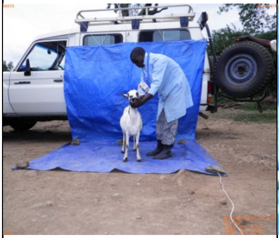	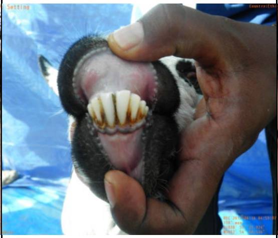	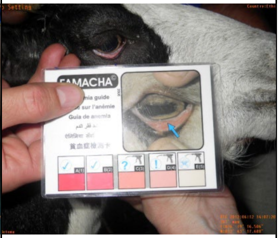
Shot 4: Front view (optional)	Shot 5: Teeth view	Shot 6: FAMACHA view

Figure 6. A complete photo series with example poses from different locations.

6. FAMACHA Shot-
    a. Pull down lower eyelid exposing conjunctiva (**Figure 6**, Shot 6)
    b. FAMACHA card (**Figure 5**). Additional FAMACHA information, included.

3. PHOTO Examples
    a. Photo Series (**Figure 7**).

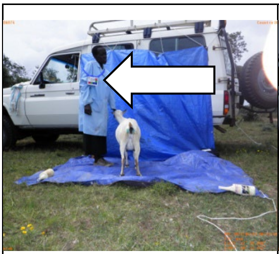 Shot 1: Rear	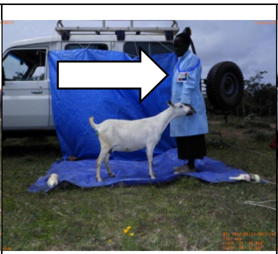 Shot 2: Side/Naked Goat	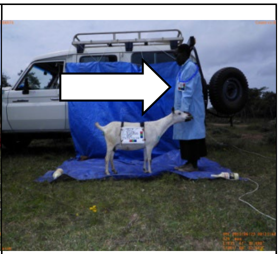 Shot 3: Sign
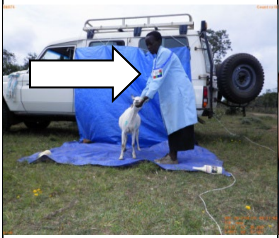 Shot 4: Front (optional)	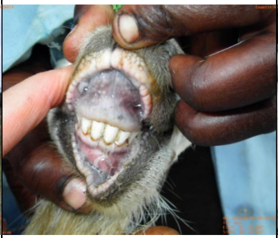 Shot 5: Teeth	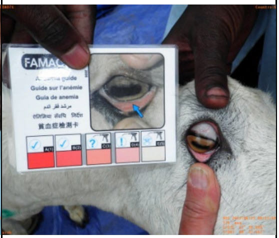 Shot 6: FAMACHA

Figure 7. Photo series of one sample goat in one location shot in Kenya, note position of small sign (arrows) in all distance photos.

b. Tips and what to look out for (**Table 1**, and **Table 2**.)

Table 1. Problems to watch out for that can make later image analysis difficult.

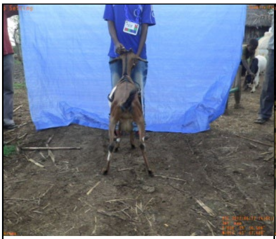	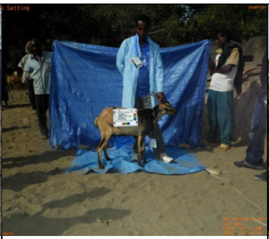	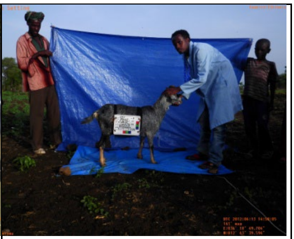
Goat lost in earth background.	Goat neck lost against pants. Sign too high, obscures backbone.	Settings: Image too dark, handler covering throat so beard cannot be visualized.
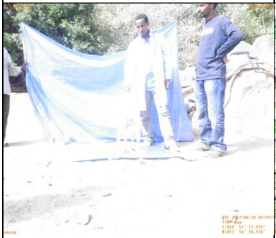	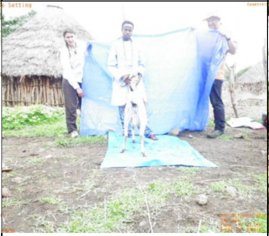	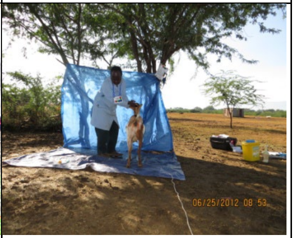
Goat lost in glare, too bright.	Camera settings: too bright.	Shadows alter backdrop color.
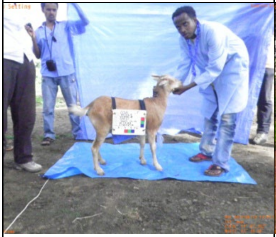	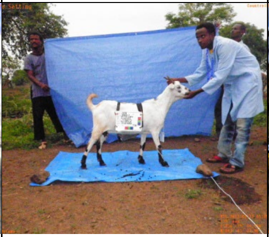	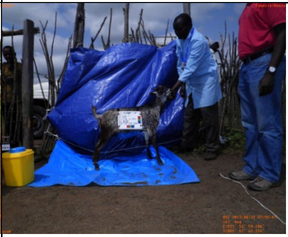
Sign sitting too low, obscures goat belly, digital measures cannot be performed directly.	Back and floor drop separated, had goat been brown, a problem to find legs.	Image too dark, backdrop folds, ripples, goat neck lost in folds.

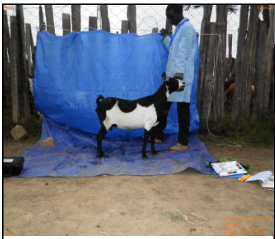	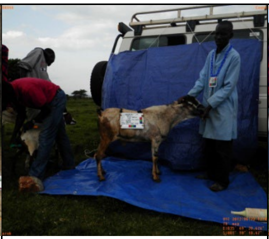	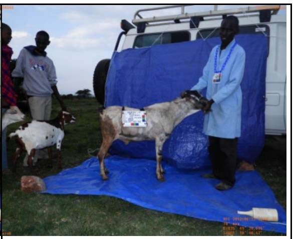
Goat neck lost in forward leg of handler.	Image too dark, and goat rump is not against blue background.	me as to left, camera ttings adjusted (+flash) mp not on backdrop.
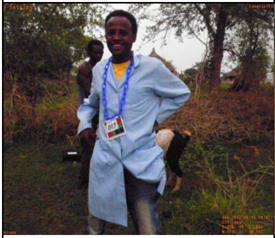	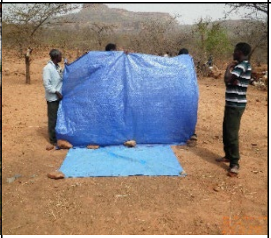	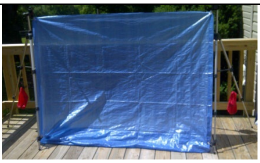 A simple backdrop stand, made of two tripods, and secured with two weights. The floor drop would be attached with clips.
A talented and cooperative handler, dressed in blue, is invaluable!	Set up showing hand-held backdrop if a stationary object is not available.	

c. FAMACHA examples and tips (**Table 2.**)

Table 2. FAMACHA examples and tips.

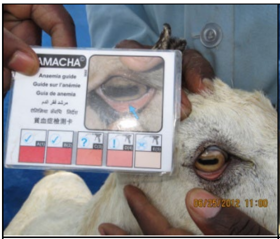	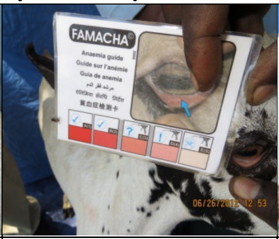	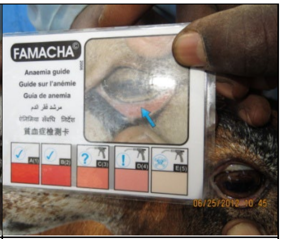
Good FAMACHA, card straight, conjunctiva well visualized, no glare on color boxes.	Good FAMACHA, card straight, conjunctiva well visualized, no glare on color boxes.	Eye too low, date covers conjunctiva, also pulling upper lid so lower cannot be pulled down.
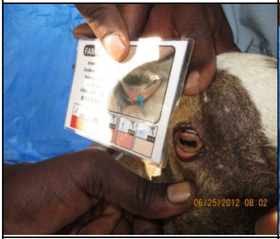	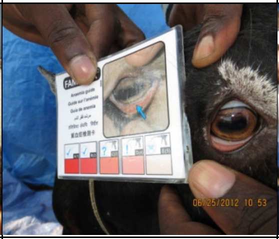	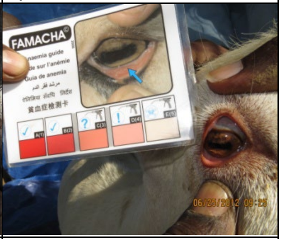
Glare obscuring FAMACHA card, card is not perpendicular to camera.	Crooked card may be harder to analyze automatically from the image.	Pulling upper lid too much, cannot see conjunctiva (lower lid).
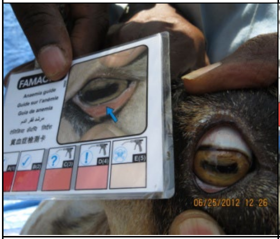	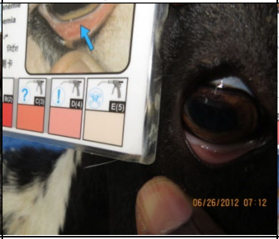	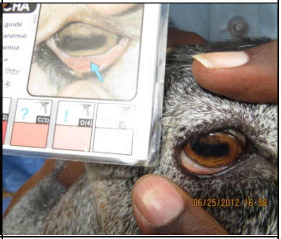
Pulling upper lid too much, cannot see conjunctiva (lower lid).	Full FAMACHA card is not in the Shot need to see all five boxes on the card, hidden conjunctiva.	Full FAMACHA card is not in the Shot need to see all five boxes on the card, hidden conjunctiva

4. BIOLOGICAL DNA SAMPLING: If you wish to collect DNA, the following protocol is provided. The collection of a biological sample from each individual goat in the form of ear tissue or whole blood to be used for subsequent genomic DNA extraction. Collect DNA via ear punch, if not possible, then blood draw, if blood draw is not possible, then do a nasal swab, if none of the above are possible, then collect hair follicles. Attempt to collect 2 samples per animal to ensure adequate DNA quality and quantity. A second sample also serves as a safeguard in case of DNA extraction failure or loss of the 1st sample. Choice of ear tissue or blood sample is based upon sampling logistics such as cold storage and shipping as well as cultural values and concerns of the farmers. Other types of biological samples such as hair follicles or nasal swabs can be used for DNA extraction. They may be taken in conjunction with ear tissue or whole blood or as an alternative to these samples. However, these biological samples (especially hair) produce lower quality and quantity of DNA which may not allow for genomic applications such as whole-genome SNP genotyping and/or whole-genome sequencing. NOTE: Make sure the blood tubes or tissue samples are labeled with goat ID. Always be prepared with antiseptic and appropriate coagulating agents to treat bleeding (specifically for handling the possibility of hitting an ear vein when using the TSUs).
    a. DNA Genotek Performagene Livestock nasal

swabs (**Figure 8**):

https://www.dnagenotek.com/US/products/collection-animals/performagene/PG-100.html

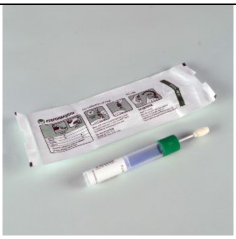

Figure 8. Genotec Performagene livestock nasal swab.

b. Blood Draw

i. Jugular vein: most practical for goats and other livestock

ii. Whole blood: 6-8ml (1ml minimum)

iii. Anti-coagulant needed to avoid clotting: Either of the following anti-coagulants works well for downstream DNA analysis. Make sure to invert tube ~6 times after blood draw to mix anti-coagulant with whole blood.

1. EDTA (Ethylenediaminetetraacetic acid)- lavender top vacutainer
2. ACD (Acid citrate dextrose)- yellow top vacutainer

iv. Storage and transport:

1. Short-term storage- whole blood should be kept cool (~4'C) after blood draw. This can be on ice packs, wet ice, or dry ice for short periods of time. It can also be stored in a refrigerator for a few days (preferably no more than 5 days).
2. Long-term storage and shipping- whole blood should be mixed by inverting the blood tube ~6 times and transferred to a plastic screwcap top cryovial appropriate for freezing. Transferring of the blood can be done by pouring directly from vacutainer to cryovial or using a sterile transfer pipette. The whole blood in cryovial storage can be frozen at negative 20'C to negative 80'C for weeks, months, etc. Any shipping of whole blood should be done on dry ice.

c. Tissue Sample

a. Tissue samples are obtained using the ALLFLEX tissue applicator system where a small ~1.5 mm diameter biopsy of ear tissue is extracted and automatically deposited in a collection unit for

preservation (Figure 9).

b. GeneMark (ALLFLEX) punch instruction guide: https://www.youtube.com/watch?v=aTVLR-nMr00

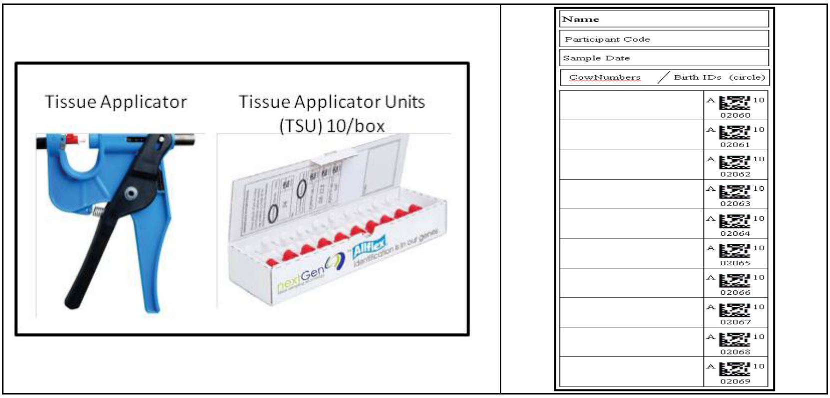

Figure 9. Tissue collection device, and sample storage (left) and barcode labels (right) for tissue sample collection.

c. Storage and Transport: The ALLFLEX TSU system comes so that the TSU can be placed back in the box at a corresponding position with the barcode on the top/front of the box. Each TSU has the last 5 digits of the barcode on the unit and a printed barcode on the bottom of the unit itself. Make sure to record the animal’s ID on the box cover with the appropriate TSU barcode. TSUs

can be stored and shipped at room temperature.[25]

d. Hair Sample

Goat Hair Sample Collection Instructions

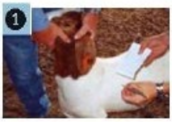

1. Check the ear tag number of the animal, and record it on the hair sample envelope. Clean the sample area to remove dirt or other contaminants.

Use bent nose, long nose or needle nose pliers to collect the sample.

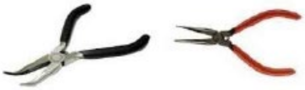

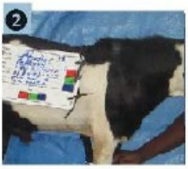

2. Pull a tuft of hair from the back of the neck or topline. Pull hair directly away from the skin, NOT at an angle. This will allow the root to come out with the hair. Take at least three pulls.

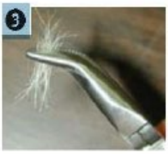

3. Inspect the hairs sample to ensure at least 40 hair follicles or roots. The roots are easy to see in goats (like human hairs with a bulb at the end), but difficult in sheep. In sheep, you may notice a color variation which indicates that the root is intact.

Note: Do NOT cut the hair from the animal. The hair MUST CONTAIN ROOTS for DNA testing. Avoid touching the roots and make sure the hair is dry

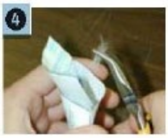

4. Place the sample in the hair sample envelope, and then seal the envelope.

Do not put hairs in a plastic bag.

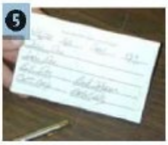

5. Fill out the remaining information lines on the envelope.

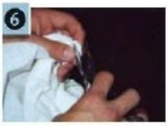

6. REMEMBER: Cleanse hands and pliers between animal samples to ensure that hairs from different animals are not mixed.

Goat Sample Checklist

- ✓ **Select collection area**
- ✓ **Record ear tag number on the envelope**
- ✓ **Obtain at least 40 hairs with follicles**
- ✓ **Take at least 3 pulls**
- ✓ **Inspect for follicles – do not touch follicles**
- ✓ **Seal envelope**
- ✓ **Clean pliers and hands between animals**

Other locations to collect hair, left and middle photos, pliers' technique on the right.

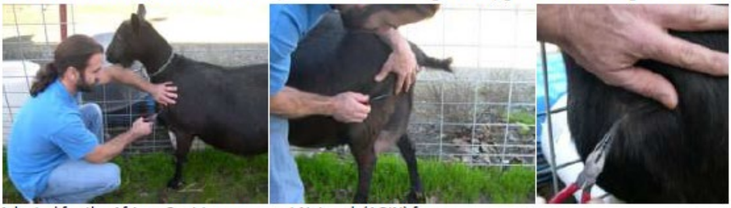

Adapted for the African Goat Improvement Network (AGIN) from:
http://igrow.org/up/articles/AnimalID_SheepGoat.pdf, and https://www.vgl.ucdavis.edu/services/goat.php.

5. PHYSICAL BODY MEASUREMENTS (**Figure 10**.) Size: IF you wish to compare and validate the digital measures, record manual (traditional) body measures as follows of:
    a. Chest (Heart) girth (CG): (body circumference at the heart, just behind the elbows, just behind the withers). ***Measure the tape when snug on the body; do not pull the tape tight***. NOTE: If you are using Caprine weigh tape, you may record BOTH circumference and weight; you MUST record circumference. This weight is a "tape weight."
    b. Height (HW): front hoof to point of withers (top of shoulder blades)
    c. Length (BL): point of shoulder to pin bone (pins=point bones on either side of anus

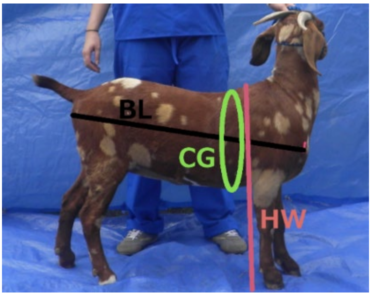

Figure 10. Chest girth (CG), height (HW), length (BL).

PHOTO KIT CONTENTS

2- six by eight-foot blue tarpaulins

4- double end bolt snaps (for connecting the two tarps (rear) at the ground where they meet)

1- 6mm x ~3 meter (~10-foot) yellow Calibration rope (with blue tape in the middle)

1- Section of yellow 6 mm x 3.5 m braided poly-pro medium load (81-pound load limit) rope to tie back drop as needed

1- Small calibration sign to place on the handler's neck- (make sure number faces the camera)

1- Large calibration sign harness to lay over the goat for the sign shots

1- Caprine weigh/measuring tape

2- DRY ERASE or WHITE BOARD markers, black to write on signs,

1- FAMACHA card

1- Blue scrubs for the handler to wear

1- Craftsman 17-inch toolbox, modified to accommodate the calibration sign harness

1- AGIN-ICP (African Goat Improvement Network Image Collection Protocol)

WHAT ELSE YOU NEED

2 to 4 - Weights to hold down the front and/or back of the ground tarp.

1- Soft cloth/dry erase board eraser to erase signs (without scratching).

NOTES OF CAUTION

1. DO NOT USE PERMANENT MARKERS or 'Sharpies' ON SIGNS, as it will permanently damage them!
2. Take care, DO NOT BEND signs as this may interfere with the image analysis.
3. The handler should stand clear of the goat (step back) so only blue tarps show behind the goats in the images.
4. The handler could wear light blue pants or skirt, shirt, gloves, and shoes.

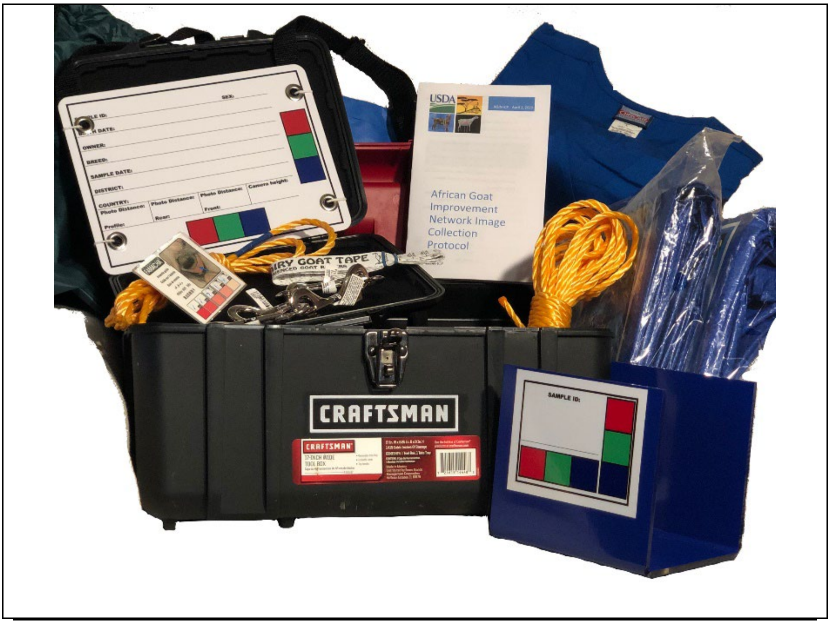

Figure 11 AGIN-ICP Sampling Equipment Kit.
