# Supplementary material for: Using the community-based breeding program (CBBP) model as a collaborative platform to develop the African Goat Improvement Network—Image collection protocol (AGIN-ICP) with mobile technology for data collection and management of livestock phenotypes
Source: Front Genet. 2023 Sep 6;14:1200770. doi: 10.3389/fgene.2023.1200770 (PMC10512022; doi:10.3389/fgene.2023.1200770)
Supplement: Supplementary file 2 [file Presentation2.pdf]

ADAPTMap SAMPLE SERIES note position of signs, handler, tarps, weights

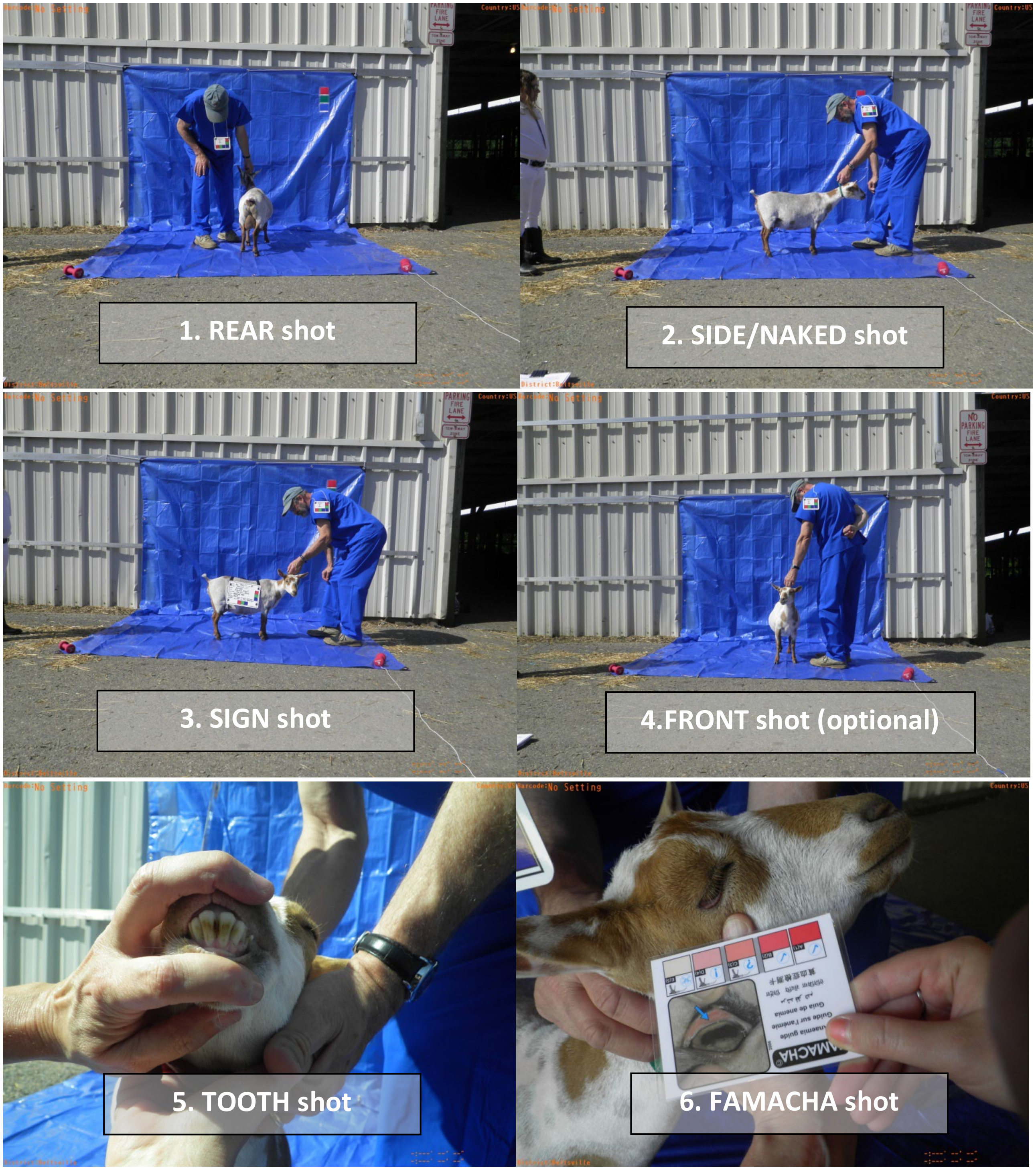

Set-up in flat, open space. Tarps connect at bottom; eliminate folds and ripples. Handler must wear blue top and bottoms. Shoot the photo series in the above sequence, camera perpendicular to goat, and at goat-eye-level. Use calibration rope to ensure camera is 3 M back from goat. Handler or other objects must not be between goat, including goat's feet/legs, and the back tarp. The small sign is visible in all distance shots. Avoid placing unnecessary objects on the tarps, and pull any lead ropes away from the goat's body.

ADAPTMap SAMPLE SERIES note position of signs, handler, tarps, weights

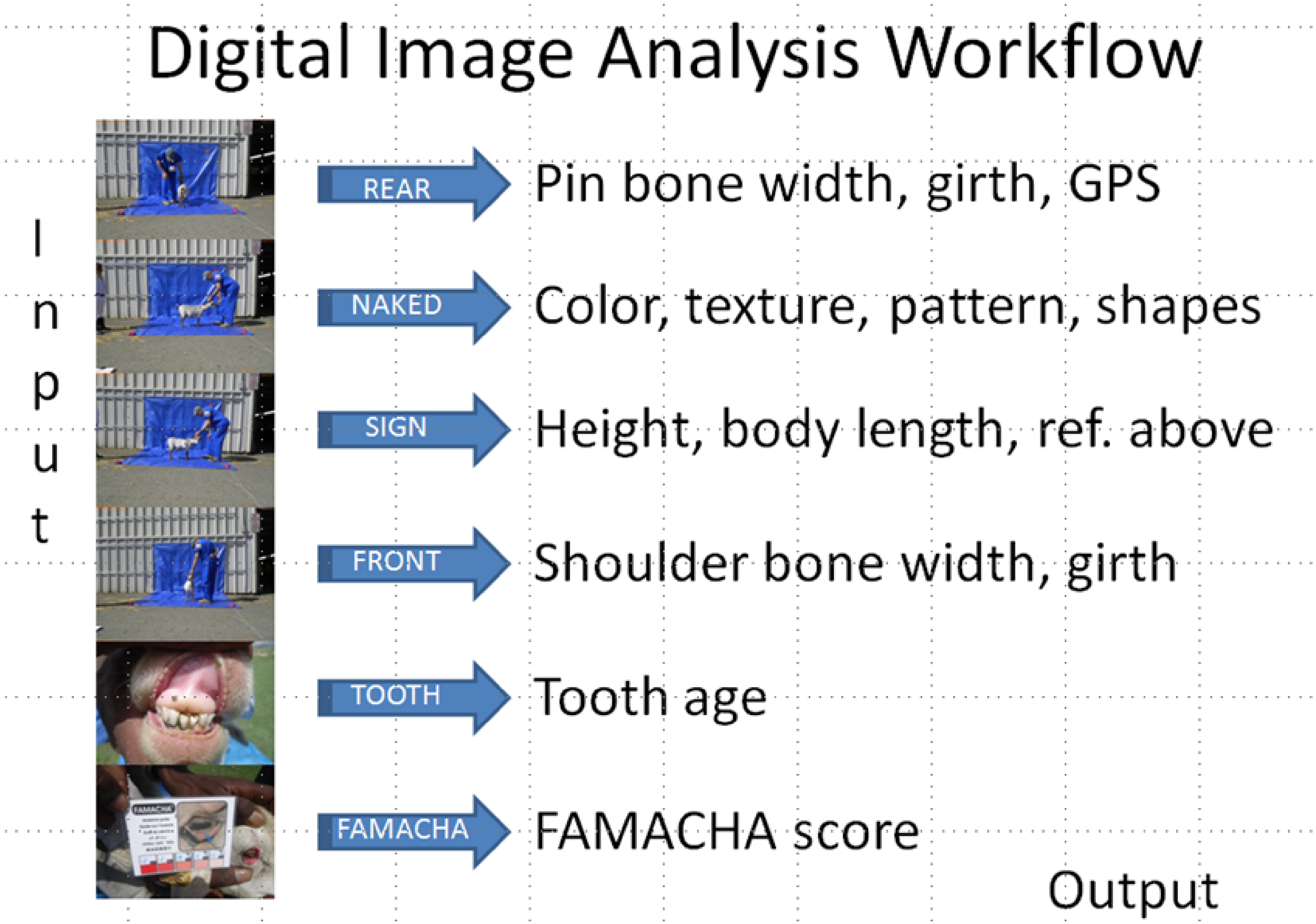

Set-up in flat, open space. Tarps connect at bottom; eliminate folds and ripples. Handler must wear blue top and bottoms. Shoot the photo series in the above sequence, camera perpendicular to goat, and at goat-eye-level. Use calibration rope to ensure camera is 3 M back from goat. Handler or other objects must not be between goat, including goat's feet/legs, and the back tarp. The small sign is visible in all distance shots. Avoid placing unnecessary objects on the tarps, and pull any lead ropes away from the goat's body.
