# Supplementary material for: Using the community-based breeding program (CBBP) model as a collaborative platform to develop the African Goat Improvement Network—Image collection protocol (AGIN-ICP) with mobile technology for data collection and management of livestock phenotypes
Source: Front Genet. 2023 Sep 6;14:1200770. doi: 10.3389/fgene.2023.1200770 (PMC10512022; doi:10.3389/fgene.2023.1200770)
Supplement: Supplementary file 3 [file Presentation3.pdf]

USDA

ADAPTMap SAMPLE SERIES position de la note de signes, de gestionnaire , des bâches, des poids

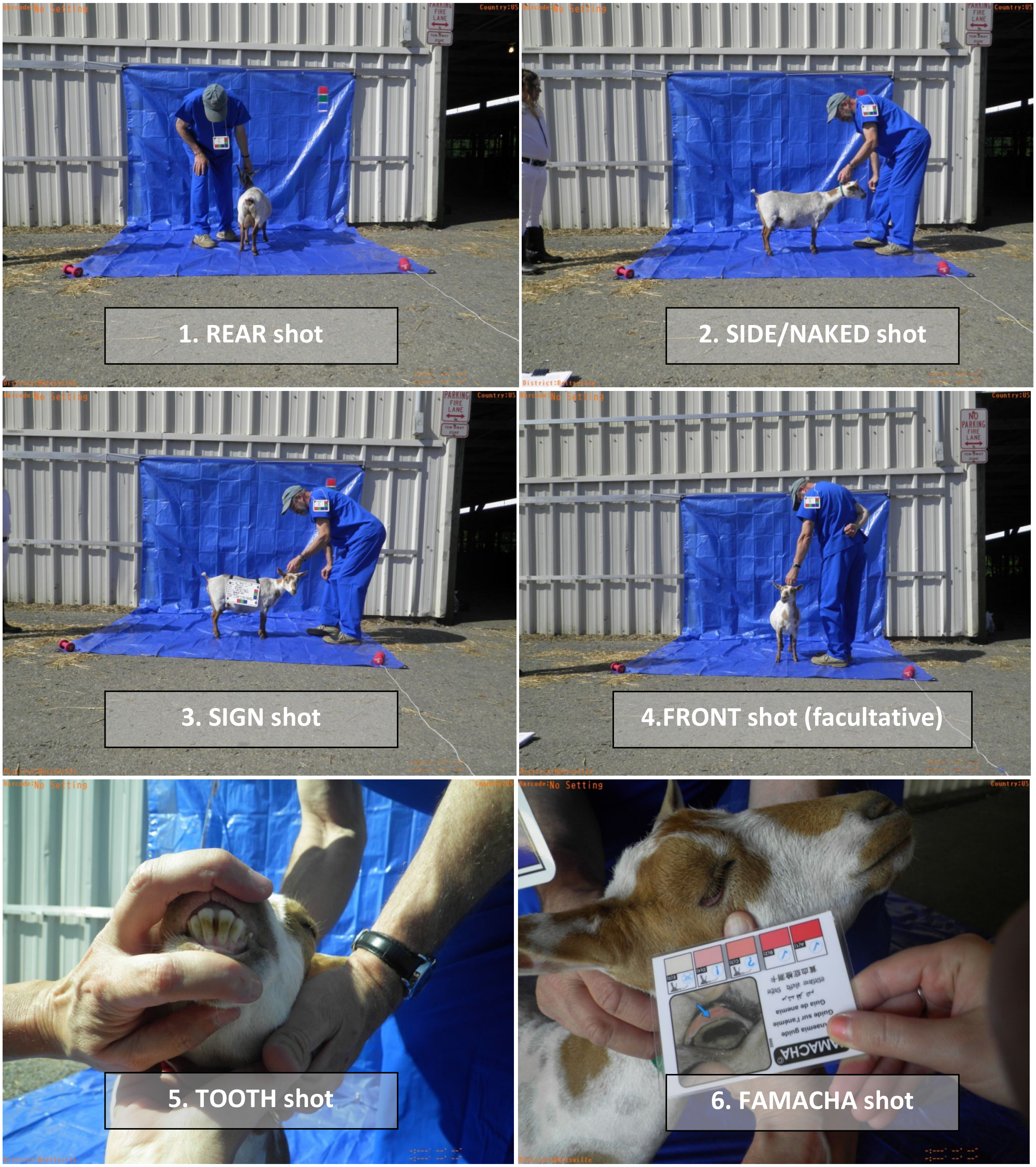

1. REAR shot

2. SIDE/NAKED shot

3. SIGN shot

4.FRONT shot (facultative)

5. TOOTH shot

6. FAMACHA shot

Set-up dans l'appartement , espace ouvert bâches connecter à fond ; éliminer les plis et des ondulations Handler doit porter haut bleu et fonds Tirez la série de photos dans la séquence ci-dessus , la caméra perpendiculaire à la chèvre et au chèvre yeux . . . niveau . Utiliser la corde d'étalonnage pour assurer la caméra est de 3 M dos de chèvre . gestionnaire ou d'autres objets ne doivent pas être entre chèvre , y compris pieds / jambes de chèvre , et l'arrière bâche . le petit signe est visible dans tous les plans de distance . Évitez de placer des objets inutiles sur les bâches , et tirer des cordes de plomb à distance du corps de la chèvre.

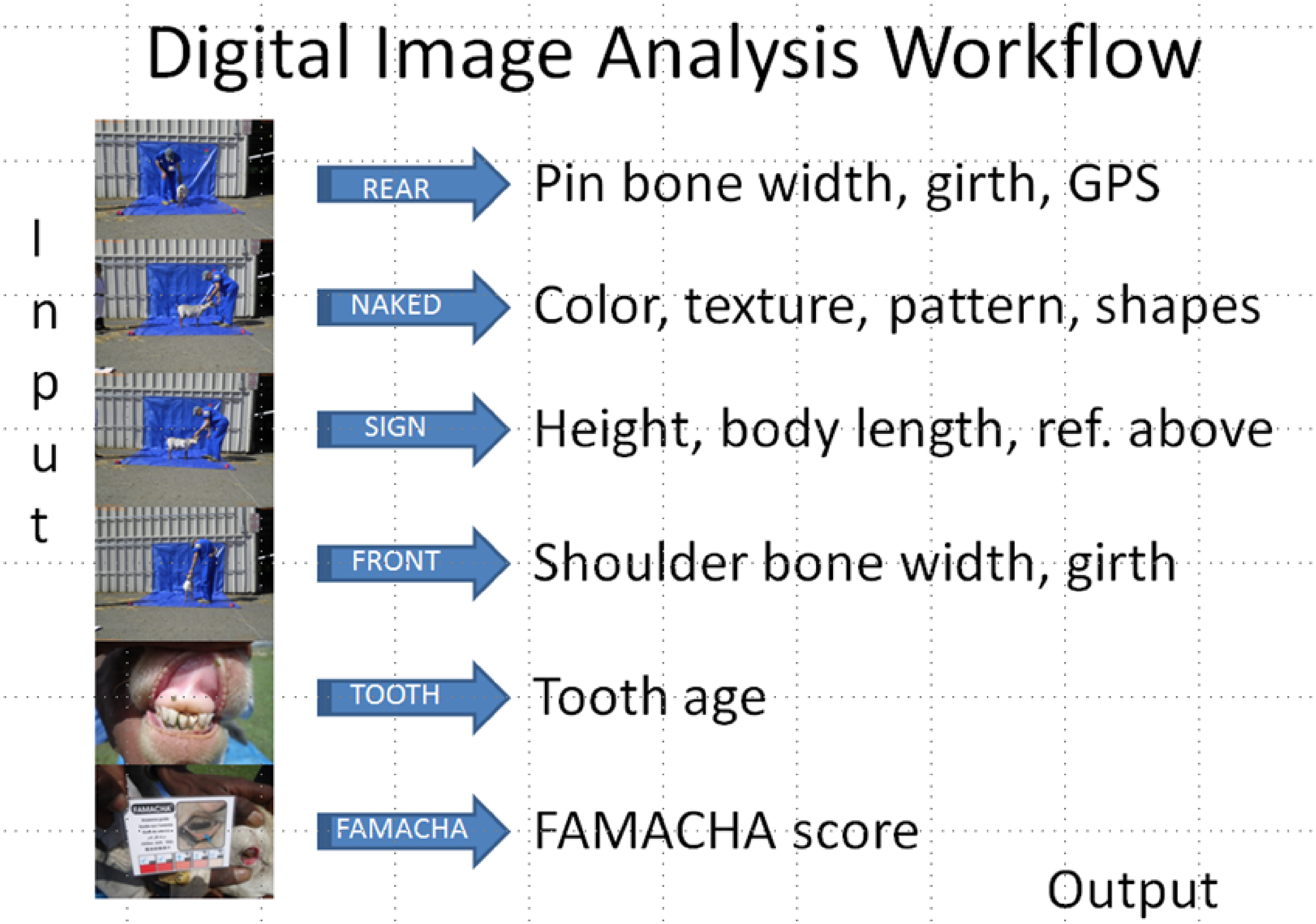

Set-up dans l'appartement , espace ouvert bâches connecter à fond ; éliminer les plis et des ondulations Handler doit porter haut bleu et fonds Tirez la série de photos dans la séquence ci-dessus , la caméra perpendiculaire à la chèvre et au chèvre yeux . . . niveau . Utiliser la corde d'étalonnage pour assurer la caméra est de 3 M dos de chèvre . gestionnaire ou d'autres objets ne doivent pas être entre chèvre , y compris pieds / jambes de chèvre , et l'arrière bâche . le petit signe est visible dans tous les plans de distance . Évitez de placer des objets inutiles sur les bâches , et tirer des cordes de plomb à distance du corps de la chèvre.
